# A Variational Bayes Genomic-Enabled Prediction Model with Genotype × Environment Interaction

**DOI:** 10.1534/g3.117.041202

**Published:** 2017-04-06

**Authors:** Osval A. Montesinos-López, Abelardo Montesinos-López, José Crossa, José Cricelio Montesinos-López, Francisco Javier Luna-Vázquez, Josafhat Salinas-Ruiz, José R. Herrera-Morales, Raymundo Buenrostro-Mariscal

**Affiliations:** * Facultad de Telemática, Universidad de Colima, 28040, México; † Departamento de Matemáticas, Centro Universitario de Ciencias Exactas e Ingenierías (CUCEI), Universidad de Guadalajara, 44430, Jalisco, México; ‡ Biometrics and Statistics Unit and Global Wheat Program of the International Maize and Wheat Improvement Center (CIMMYT), 06600 México D.F., México; § Departamento de Estadística, Centro de Investigación en Matemáticas (CIMAT), 36240 Guanajuato, México; ** Colegio de Postgraduados, Campus Córdoba, Km. 348 Carretera Federal Córdoba-Veracruz, Amatlán de los Reyes, 94946, México

**Keywords:** variational Bayes, multi-environment, genome-enabled prediction, GenPred, Shared Data Resources, Genomic Selection

## Abstract

There are Bayesian and non-Bayesian genomic models that take into account G×E interactions. However, the computational cost of implementing Bayesian models is high, and becomes almost impossible when the number of genotypes, environments, and traits is very large, while, in non-Bayesian models, there are often important and unsolved convergence problems. The variational Bayes method is popular in machine learning, and, by approximating the probability distributions through optimization, it tends to be faster than Markov Chain Monte Carlo methods. For this reason, in this paper, we propose a new genomic variational Bayes version of the Bayesian genomic model with G×E using half-t priors on each standard deviation (SD) term to guarantee highly noninformative and posterior inferences that are not sensitive to the choice of hyper-parameters. We show the complete theoretical derivation of the full conditional and the variational posterior distributions, and their implementations. We used eight experimental genomic maize and wheat data sets to illustrate the new proposed variational Bayes approximation, and compared its predictions and implementation time with a standard Bayesian genomic model with G×E. Results indicated that prediction accuracies are slightly higher in the standard Bayesian model with G×E than in its variational counterpart, but, in terms of computation time, the variational Bayes genomic model with G×E is, in general, 10 times faster than the conventional Bayesian genomic model with G×E. For this reason, the proposed model may be a useful tool for researchers who need to predict and select genotypes in several environments.

Nowadays, genomic-assisted selection (GS) is a very attractive breeding tool for selecting candidate genotypes early, using only dense molecular markers without the need to phenotype the validation population for several complex traits. However, to take advantage of all the available information (markers, multiple-traits, multiple-environments, etc.), more efficient statistical methods are desirable for genome-enabled prediction models. Markov Chain Monte Carlo (MCMC) methods provide an effective means of dealing with complex models, and are widely used in genomic prediction models because they allow simulation of high-dimensional distributions to arbitrary levels of accuracy. However, MCMC methods are computationally expensive, especially in the presence of intractable multivariate integrals in posterior densities and likelihood (Ormerod and Wand 2012).

For the above reasons, the variational Bayes method (VBM), which is very popular in computer science and the machine learning community, has been adopted by the statistical science community, because, although variational approximations kill some accuracy of the MCMC methods, they offer vast improvements in terms of computational speed and time. The VBM facilitates approximate inference for the parameters in complex statistical models, and provides fast, deterministic alternatives to Monte Carlo methods (Ormerod and Wand 2010). Most VBM applications are found in the machine learning community, and there is enough empirical evidence that the VBM tends to be faster than classic methods such as MCMC sampling. Also, this method is easier to scale to large data—it has been applied to problems such as large-scale document analysis, high-dimensional neuroscience, and computer vision, as well as in computational biology, robotics, speech recognition, marketing, optimal control, reinforcement learning, statistical network analysis, astrophysics, and the social sciences (Blei *et al.* 2016). However, the VBM has been studied less rigorously than MCMC, and its statistical properties are less understood (Blei *et al.* 2016).

In GS and prediction, we have found some applications for the VBM, for: (1) clustering analysis of gene-expression data (Teschendorff *et al.* 2005); (2) conducting genome-wide association studies (Logsdon *et al.* 2010; Carbonetto and Stephens 2012); (3) predicting genomic breeding values in univariate and multiple traits (Hayashi and Iwata 2013; Yamamoto *et al.* 2017); (4) estimating quantitative trait locus effects (Li and Sillanpää 2012, 2013), and (5) estimating variance components in the context of animal science (Arakawa *et al.* 2016).

Most VBMs applied in genomic-enabled prediction use a simple linear model of the form $y_{i}=\mu +\sum_{j=1}^{m}x_{ij}\beta_{j}+\varepsilon_{i},$ where $y_{i}$ is the phenotype of the ith individual, μ is the sample mean, $x_{ij}$ defines the genotype of the jth marker, and $\varepsilon_{i}$ is an error term normally distributed with mean zero and variance $\sigma_{\varepsilon }^{2}$ (Logsdon *et al.* 2010; Carbonetto and Stephens 2012; Yamamoto *et al.* 2017; Li and Sillanpää 2012; Teschendorff *et al.* 2005). Exceptions to the use of simple linear models in VBM were studied by Hayashi and Iwata (2013), who modeled multiple traits without effects of environments and without interaction terms; by Arakawa *et al.* (2016), who used a model for univariate responses with mixed effects without interaction terms; and in the research of Li and Sillanpää (2013), who used a model for functional regression without random effects and without interaction terms.

Based on the above, the purpose of this study was to propose a univariate genomic VBM that takes into account genotype × environment interaction (G×E), and could be an alternative to the existing Bayesian and non-Bayesian models with G×E described by Jarquín *et al.* (2014) in the reaction norm model. Since animal and plant breeding programs today perform genetic evaluations of large numbers of lines, or animals in many environments, for several traits and genotyped with thousands (or millions) of genetic markers, complex Bayesian models may take days or weeks to do this, and, as a result, the existing models become untenable.

For this reason, in order to retain all the useful features of the Bayesian genomic models with G×E at a reduced computational cost, we propose, within the general framework of variational Bayes, a method for estimating the univariate genomic model with G×E, which we call the variational Bayes multi-environment (VBME) model. This means that the VBME will recast the problem of computing posterior densities by the Gibbs sampler of the conventional Bayesian methods with G×E—referred to here as the Bayesian multi-environment (BME) model—as an optimization problem using the VBM, which consists of introducing a class of approximating distributions of the latent variables, then optimizing some criteria to find the distribution within this class that best matches the posterior (Carbonetto and Stephens 2012). Also, it is important to point out that the newly proposed method uses, as a prior for all standard deviations (SDs), a half-t distribution that is a high noninformative prior distribution (Huang and Wand 2013). We derived the full conditional and variational posterior distribution of the VBME model, explained its implementation, and applied it to eight experimental genomic (maize and wheat) data sets with two main objectives: (1) to compare the accuracy of the VBME with that of the standard BME model; and (2) to compare the computational processing time of VBME with that of BME.

## Materials and Methods

### The variational Bayes method using the density transform approach

The most common variants of the VBM are the density transform approach and the tangent transform approach. We will explain the basis of the density transform approach; readers interested in understanding the tangent transform approach can consult Ormerod and Wand (2010). The density transform approach consists of approximating the posterior densities through other densities for which inference is more tractable. In Bayesian statistics, the inference problem is to compute the conditional distribution of unknown parameters given the observations *p*(***θ***│***y***) = *p*(***y***,***θ***)/*p*(***y***); the denominator is known as the marginal distribution of the observations or model evidence, ***θ***∈**Θ** is the parameter vector, and p(y,θ) is the joint likelihood of the data and model parameters. The essence of the density transform approach is to approximate the posterior density p(θ/y) by a function q(θ), for which the *q*-dependent lower bound [p(y;q)]on the marginal likelihood is given by

$$\underline{p}\left(y;q\right)\equiv \exp\int q\left(\theta \right)\log\left(\frac{p\left(y,\theta \right)}{q\left(\theta \right)}\right)d\theta$$ (1)

Expression (1) is more tractable than the marginal likelihood, p(y). Indeed, the maximization of $\underline{p}\left(y;q\right)$ with respect to q is equivalent to minimizing the Kullback-Leibler divergence between q(θ) and p(θ/y), KL(q(⋅),p(⋅/y)). This is because the marginal likelihood satisfies

$$p\left(y\right)=\exp\left(\mathrm{KL}\left(q\left(\cdot \right),p\left(\cdot |y\right)\right)\right)\underline{p}\left(y;q\right)\geq \underline{p}\left(y;q\right)$$ (2)

which can be derived through Jensen’s inequality, and the equality holds if, and only if, q(θ)=p(θ/y) almost everywhere (Ormerod and Wand 2010). To achieve tractability, $\underline{p}\left(y;q\right)$ is maximized over a restricted, more manageable, q class of densities.

Here, for the implementation, we chose, as restrictions for the q class of densities, the *mean field variational family restriction*, where the latent variables are mutually independent, and each is governed by a distinct factor in the variational distribution, that is, q(θ) factorizes into $\prod_{j=1}^{m}q_{j}\left(\theta_{j}\right)$ for some partitions $\left\{\theta_{1},\ldots ,\theta_{m}\right\}$ of θ. Each $\theta_{j}$ is governed by its own distribution $q_{j}\left(\theta_{j}\right)$, and, in principle, each variational factor can take on any parametric form that is appropriate for the corresponding random variable. In the optimization process, these variational factors are chosen to maximize the evidence lower bound given in Equation (2). One advantage of using the mean field variational family restriction over q(θ) is that it is possible to obtain explicit solutions for each product component in terms of the others when conjugate priors are used. These lead to an interactive scheme for obtaining simultaneous solutions. The solutions rely on result 2.1.

Result. 2.1. Let v and w be continuous random vectors with joint density p(v,w). Then

$$\sup_{q}\left\{\int q\left(v\right)\log\left(\frac{p\left(v,w\right)}{q\left(v\right)}\right)dv\right\}$$

is attained by q∗(v)=p(v|w).

One disadvantage of the mean field variational family restriction is its tendency to underestimate the variability of parameter estimates; it is also not a good option when the parameters under study have a high degree of dependence, which causes degradation in the resulting inference. Conversely, if the posterior dependence between parameters is weak, then the product density restriction could lead to very accurate approximate inferences (Ormerod and Wand 2010; Blei *et al.* 2016). When conditional conjugate priors are used, given the other variational factor components, the optimal densities of each variational factor, $q_{j}\left(\theta_{j}\right),$ are proportional to the exponentiated expected log of the full conditionals obtained with Gibbs sampling, that is,

$$q_{j}\left(\theta_{j}\right)\propto \exp\left\{E_{-\theta_{j}}\log\;p\left(\theta_{j}|ELSE\right)\right\}$$ (3)

where $p\left(\theta_{j}|ELSE\right)$ are the known full conditionals in the MCMC literature, and the expectations on the right-hand side of Equation (3) are computed with respect to the density *q*(***θ***)/*q*(***θ****_j_*). From this equation, an interactive scheme can be used to obtain the optimal densities. Equation (3) allows obtaining all the variational posteriors for implementing the VBM using mean variational approximation.

### Statistical model

Since we wanted to develop a variational Bayes version of the BME model, we used $y_{ijk}$ to represent the normal phenotype from the kth replication of the jth line in the ith environment (i=1,2,…I, j=1,2,…,J, k=1,2,…,K), where K represents the number of replicates of each line in each environment under study. Therefore, the total number of observations is n=I×J×K. Therefore, the BME model is

$$y_{ijk}=E_{i}+g_{j}+gE_{ij}+e_{ijk}$$ (4)

where $E_{i}$ represents the effect of ith environment, $g_{i}$ represents the genomic effect of jth line, and is assumed as a random effect, $gE_{ij}$ is the interaction between the genomic effect of the jth line and the ith environment, and is assumed a random effect, and $e_{ijk}$ is a random error term associated with the kth replication of the jth line in the ith environment.

In matrix notation, the model given in Equation (4) including all the information is expressed as:

$$Y=X\beta +Z_{1}b_{1}+Z_{2}b_{2}+e$$ (5)

where Y is the response vector of order n×J, X is the design matrix of environment effects of order n×I, β is the regression coefficient vector of order I×1 associated with the environment effects, $Z_{1}$ is the design matrix of order n×J, associated with the random effects of lines, $b_{1}$ is the vector of random effects of lines of order J×1, $Z_{2}$ is the design matrix of order n×IJ, associated with the interaction random effects between lines and environments, $b_{2}$ is the vector of interaction random effects of lines and environments of order IJ×1, and e is the vector of random error terms of order n×1. Then, we assumed that $b_{1}∼N\left(0,G\sigma_{1}^{2}\right),$ $b_{2}∼N\left(0,G_{2}\sigma_{2}^{2}\right)$, and $e∼N\left(0,I_{n}\sigma_{e}^{2}\right),$ also that this vector is statistically independent, where G denotes the genomic relationship matrix, and was calculated as suggested by VanRaden (2008). $G_{2}=I_{I}⊗G,$ ⊗ denotes a Kronecker product, where $I_{I}$ is an identity matrix of order I×I, which indicates that we are assuming independence between environments.

### BME model

The joint posterior density of the parameter vector of the BME model is given by

$$\begin{array}{l} p\left(\beta ,b_{1},b_{2},\sigma_{1}^{2},\sigma_{2}^{2},\sigma_{\beta }^{2},\sigma_{e}^{2},a_{\beta },a_{b1},a_{b2},a_{e}\right)\\ \propto p\left(Y|\beta ,b_{1},b_{2},\sigma_{e}^{2}\right)p\left(\beta |\sigma_{\beta }^{2}\right)p\left(\sigma_{\beta }^{2}|a_{\beta }\right)p\left(a_{\beta }\right)p\left(b_{1}|\sigma_{1}^{2}\right)p\left(\sigma_{1}^{2}|a_{b1}\right)\times p\left(a_{b1}\right)\\ p\left(b_{2}|\sigma_{2}^{2}\right)p\left(\sigma_{2}^{2}|a_{b2}\right)p\left(a_{b2}\right)p\left(\sigma_{e}^{2}|a_{e}\right)p\left(a_{e}\right) \end{array}$$ (6)

which is not a known distribution that is easy to simulate, so we will use MCMC techniques to obtain samples of this by means of the Gibbs sampler. The notation x∼IG(A,B) represents a random variable, x, that has an inverse Gamma distribution with shape A and scale B parameters A,B>0, with density function $P\left(x\right)=B^{A}\Gamma {\left(A\right)}^{-1}x^{-A-1}\exp\left[-\left(B/x\right)\right],$ x>0. The following prior specification is given:

$$\begin{array}{l} \beta |\sigma_{\beta }^{2}∼N_{p}\left(\beta_{\beta },\Sigma_{\beta }\sigma_{\beta }^{2}\right),\sigma_{\beta }^{2}|a_{\beta }∼IG\left(\nu_{\beta }/2,\nu_{\beta }/a_{\beta }\right),a_{\beta }∼IG\left(1/2,1/A_{\beta }^{2}\right),\\ \sigma_{1}^{2}|a_{b_{1}}∼IG\left(\nu_{b_{1}}/2,\nu_{b_{1}}/a_{b_{1}}\right),a_{b_{1}}∼IG\left(1/2,1/A_{l}^{2}\right),\sigma_{e}^{2}|a_{e}∼IG\left(\nu_{e}/2,\nu_{e}/a_{e}\right),\\ a_{e}∼IG\left(1/2,1/A_{e}^{2}\right),\sigma_{2}^{2}|a_{b2}∼IG\left(\nu_{b2}/2,\nu_{b2}/a_{b_{2}}\right)\text{and}\;a_{b2}∼IG\left(1/2,1/A_{2}^{2}\right). \end{array}$$

It is important to point out that the hierarchical priors given to the SDs induce Half-t priors to achieve arbitrary high noninformativity in this parameter (Huang and Wand 2013). Note that the use of this type of priors of our proposed model for genomic-enabled prediction is different than the priors used in the context of the existing Bayesian univariate models with G×E. Details of all full conditional distributions derived from the Gibbs sampler for implementing these BME and VBME models are given in Appendices A and B, whereas the R code for implementing the VBME model is provided in Appendix D.

### Variational version of the BME model

As an alternative to the Gibbs sampler for estimating the parameters, in this section, we will derive the optimal densities under the mean field variational family restriction, assuming the following factorization of q(θ):

$$\begin{array}{l} q\left(\beta ,b_{1},b_{2},\sigma_{b_{1}}^{2},\sigma_{b_{2}}^{2},\sigma_{\beta }^{2},\sigma_{e}^{2},a_{\beta },a_{b_{1}},a_{b_{2}},a_{e}\right)\\ =q\left(\beta \right)q\left(b_{1}\right)q\left(b_{2}\right)q\left(\sigma_{b_{1}}^{2}\right)q\left(\sigma_{b_{2}}^{2}\right)q\left(\sigma_{\beta }^{2}\right)q\left(\sigma_{e}^{2}\right)q\left(a_{\beta }\right)q\left(a_{b_{1}}\right)q\left(a_{b_{2}}\right)q\left(a_{e}\right) \end{array}$$ (7)

The difficult problem of finding the exact posterior (6) became less difficult, *i.e.*, it now consists of finding an approximate parametric posterior $q\left(\beta ,b_{1},b_{2},\sigma_{1}^{2},\sigma_{2}^{2},\sigma_{\beta }^{2},\sigma_{e}^{2},a_{\beta },a_{b1},a_{b2},a_{e}\right)$ with moments (*i.e.*, parameters). Inference then reduces to finding a density q that minimizes a measure of dissimilarity between q and p. This can be achieved by maximizing the log of the evidence lower bound that is an approximation of the BME model using Equation (6) with respect to (the moments of) q. For details, see MacKay (1995), Attias (2000), Ghahramani and Beal (2001) and Bishop (1999).

Because in the Bayesian formulation of the model given in Equation (5), we use conditional conjugate priors over parameters of the corresponding partition of θ, in the factorization assumed for q(θ) in Equation (7), all the optimal distributions (Equation 3) of the VBME model of each variational factor, $q_{j}\left(\theta_{j}\right),$ belong to the same family as the full conditional distributions for the BME model. Because the same Bayesian model (BME) was converted to its variational counterpart (VBME), both models are comparable. The derivation details of all these densities are given in Appendix A.

In summary, the iterative scheme used to obtain the optimal density  q(θ) over the class of densities given in Equation (7), is given by the following algorithm. Before presenting the algorithm, we provide some basic notations. We will use $\mu_{q}\left(\theta_{j}\right)$ and $\sigma_{q}^{2}\left(\theta_{j}\right)$ to denote the mean and variance of $\;q_{j}$ distribution. For vector parameter θ, we use the analogously defined $\mu_{q}\left(\theta \right)$ and $\Sigma_{q\left(\theta \right)}.$ Therefore, the expected values for the inverse of the parameters $\sigma_{\beta }^{2},a_{\beta },\sigma_{1}^{2},a_{b1},\sigma_{2}^{2},a_{b2},\sigma_{e}^{2},\;\text{and}\;a_{e}$ used were: $\mu_{q\left(1/\sigma_{\beta }^{2}\right)}=\left[0.5\left(\nu_{\beta }+I\right)/\left(B_{q\left(\sigma_{\beta }^{2}\right)}\right)\right],$ $\mu_{q\left(1/a_{\beta }\right)}=\left[0.5\left(\nu_{\beta }+1\right)/\left(B_{q\left(a_{\beta }\right)}\right)\right],\;\mu_{q\left(1/\sigma_{1}^{2}\right)}=\left[0.5\left(\nu_{b_{1}}+J\right)/\left(B_{q\left(\sigma_{1}^{2}\right)}\right)\right],$ $\mu_{q\left(1/a_{b_{1}}\right)}=\left[0.5\left(\nu_{b_{1}}+1\right)/\left(B_{q\left(a_{b_{1}}\right)}\right)\right],$ $\mu_{q\left(1/\sigma_{2}^{2}\right)}=\left[0.5\left(\nu_{b_{2}}+IJ\right)/\left(B_{q\left(\sigma_{2}^{2}\right)}\right)\right],$ $\mu_{q\left(1/a_{b_{2}}\right)}=\left[0.5\left(\nu_{b_{2}}+1\right)/\left(B_{q\left(a_{b_{2}}\right)}\right)\right],$ $\mu_{q\left(1/\sigma_{e}^{2}\right)}=\left[0.5\left(\nu_{e}+n\right)/\left(B_{q\left(\sigma_{e}^{2}\right)}\right)\right],$ and $\mu_{q\left(1/a_{e}\right)}=\left[0.5\left(\nu_{e}+1\right)/\left(B_{q\left(a_{e}\right)}\right)\right],$ respectively, while, for the scale parameters, we will use the notation $B_{q}\left(\theta_{j}\right).$ Therefore, $B_{q\left(\sigma_{\beta }^{2}\right)},B_{q\left(a_{\beta }\right)},$$B_{q\left(\sigma_{1}^{2}\right)},B_{q\left(a_{b1}\right)},B_{q\left(\sigma_{2}^{2}\right)},B_{q\left(a_{b2}\right)},B_{q\left(\sigma_{e}^{2}\right)},$ and $B_{q\left(a_{e}\right)}$ represent the scale parameters of the variational posterior distribution of $\sigma_{\beta }^{2},a_{\beta },\sigma_{1}^{2},a_{b1},\sigma_{2}^{2},a_{b2},\sigma_{e}^{2}$, and $a_{e},$ respectively. Also, $e*=Y-X\mu_{q\left(\beta \right)}-Z_{1}\mu_{q\left(b_{1}\right)}-Z_{2}\mu_{q\left(b_{2}\right)}$, and $\beta_{\beta }$ is the prior of the beta regression coefficients.

### Algorithm for implementing the VBME

Initialize: $\mu_{q\left(\beta \right)}=0,$ $\mu_{q\left(b_{1}\right)}=0,$ $\mu_{q\left(b_{2}\right)}=0,$ $\Sigma_{q\left(b_{1}\right)}=I_{J},$ $\Sigma_{q\left(b_{2}\right)}=I_{IJ},$ $B_{q\left(\sigma_{\beta }^{2}\right)},B_{q\left(a_{\beta }\right)},B_{q\left(\sigma_{1}^{2}\right)},B_{q\left(a_{b1}\right)},B_{q\left(\sigma_{2}^{2}\right)},B_{q\left(a_{b2}\right)},\;B_{q\left(\sigma_{e}^{2}\right)},B_{q\left(a_{e}\right)}>0.$Cycle:

$$\Sigma_{q\left(\beta \right)}\leftarrow {\left(\Sigma_{\beta }^{-1}\mu_{q\left(1/\sigma_{\beta }^{2}\right)}+X^{T}\left(I_{n}\mu_{q\left(\sigma_{e}^{-2}\right)}\right)X\right)}^{-1}$$

$$\mu_{q\left(\beta \right)}\leftarrow \Sigma_{q\left(\beta \right)}\left(\Sigma_{\beta }^{-1}\beta_{\beta }\mu_{q\left(1/\sigma_{\beta }^{2}\right)}+X^{T}\left[I_{n}\mu_{q\left(\sigma_{e}^{-2}\right)}\right]\left[Y-\sum_{h=1}^{2}Z_{h}\mu_{q\left(b_{h}\right)}\right]\right)$$

$$B_{q\left(\sigma_{\beta }^{2}\right)}\leftarrow \frac{{\left[\mu_{q\left(\beta \right)}-\beta_{\beta }\right]}^{T}\Sigma_{\beta }^{-1}\left[\mu_{q\left(\beta \right)}-\beta_{\beta }\right]+tr\left(\Sigma_{\beta }^{-1}\Sigma_{q\left(\beta \right)}\right)}{2}+\nu_{\beta }\mu_{q\left(1/a_{\beta }\right)}$$

$$B_{q\left(a_{\beta }\right)}\leftarrow 1/A_{\beta }^{2}+\nu_{\beta }\mu_{q\left(1/\sigma_{\beta }^{2}\right)}$$

$$\Sigma_{q\left(b_{1}\right)}\leftarrow {\left(G^{-1}\mu_{q\left(\sigma_{1}^{-2}\right)}+Z_{1}^{T}\left[I_{n}\mu_{q\left(\sigma_{e}^{-2}\right)}\right]Z_{1}\right)}^{-1}$$

$$\mu_{q\left(b_{1}\right)}\leftarrow \Sigma_{q\left(b_{1}\right)}\left(Z_{1}^{T}\left[I_{n}\mu_{q\left(\sigma_{e}^{-2}\right)}\right]\left[Y-X\mu_{q\left(\beta \right)}-Z_{2}\mu_{q\left(b_{2}\right)}\right]\right)$$

$$\Sigma_{q\left(b_{2}\right)}\leftarrow {\left(G_{2}^{-1}\mu_{q\left(\sigma_{2}^{-2}\right)}+Z_{2}^{T}\left[I_{n}\mu_{q\left(\sigma_{e}^{-2}\right)}\right]Z_{2}\right)}^{-1}$$

$$\mu_{q\left(b_{2}\right)}\leftarrow \Sigma_{q\left(b_{2}\right)}\left(Z_{2}^{T}\left[I_{n}\mu_{q\left(\sigma_{e}^{-2}\right)}\right]\left[Y-X\mu_{q\left(\beta \right)}-Z_{1}\mu_{q\left(b_{1}\right)}\right]\right)$$

$$B_{q\left(\sigma_{1}^{2}\right)}\leftarrow \frac{\mu_{q\left(b_{1}\right)}^{T}G^{-1}\mu_{q\left(b_{1}\right)}+tr\left(G^{-1}\Sigma_{q\left(b_{1}\right)}\right)}{2}+\nu_{b_{1}}\mu_{q\left({\frac{1}{a}}_{b_{1}}\right)}$$

$$B_{q\left(a_{b_{1}}\right)}\leftarrow 1/A_{b_{1}}^{2}+\nu_{b_{1}}\mu_{q\left(\frac{1}{\sigma_{b_{1}}^{2}}\right)}$$

$$B_{q\left(\sigma_{2}^{2}\right)}\leftarrow \frac{\mu_{q\left(b_{2}\right)}^{T}G_{2}^{-1}\mu_{q\left(b_{2}\right)}+tr\left(G_{2}^{-1}\Sigma_{q\left(b_{2}\right)}\right)}{2}+\nu_{b_{2}}\mu_{q\left(1/a_{b_{2}}\right)}$$

$$B_{q\left(a_{b_{2}}\right)}\leftarrow 1/A_{b_{2}}^{2}+\nu_{b_{2}}\mu_{q\left(1/\sigma_{2}^{2}\right)}$$

$$B_{q\left(\sigma_{e}^{2}\right)}\leftarrow \frac{e^{*T}e*+tr\left(X^{t}X\Sigma_{q\left(\beta \right)}\right)+tr\left(Z_{1}^{T}Z_{1}\Sigma_{q\left(b_{1}\right)}\right)+tr\left(Z_{2}^{T}Z_{2}\Sigma_{q\left(b_{1}\right)}\right)}{2}+\nu_{e}\mu_{q\left(\frac{1}{a_{e\;}}\right)}$$

$$B_{q\left(a_{e}\right)}\leftarrow \frac{1}{A_{e}^{2}}+\nu_{e}\mu_{q\left(\sigma_{e}^{-2}\right)}$$

until the change in the lower bound, $\log\underline{p}\left(y;q\right),$ between iterations is less than a tolerance value specified *a priori*. For our choice of approximating distribution (see Appendix B), in each cycle of the algorithm, $\log\left(\underline{p}\left(y;q\right)\right)$ has the following analytical expression:

$$\log\underline{p}\left(y;q\right)=0.5\log\left(\left|\Sigma_{q\left(\beta \right)}\right|\right)-0.5\left(\nu_{\beta }+I\right)\log\left(B_{q\left(\sigma_{\beta }^{2}\right)}\right)-0.5\left(\nu_{\beta }+1\right)\log\left(B_{q\left(a_{\beta }\right)}\right)+\;\mu_{q\left(\frac{1}{a_{\beta }}\right)}\nu_{\beta }\mu_{q\left(\frac{1}{\sigma_{\beta }^{2}}\right)}+0.5\log\left(\left|\Sigma_{q\left(b_{1}\right)}\right|\right)+0.5\log\left(\left|\Sigma_{q\left(b_{2}\right)}\right|\right)-0.5\left(\nu_{b1}+J\right)\log\left(\left|B_{q\left(\sigma_{1}^{2}\right)}\right|\right)-0.5\left(\nu_{b1}+1\right)\log\left(B_{q\left(a_{b1}\right)}\right)+\mu_{q\left(\frac{1}{a_{b1}}\right)}\nu_{b1}\mu_{q\left(\frac{1}{\sigma_{1}^{2}}\right)}-0.5\left(\nu_{b2}+IJ\right)\log\left(\left|B_{q\left(\sigma_{2}^{2}\right)}\right|\right)-0.5\left(\nu_{b2}+1\right)\log\left(B_{q\left(a_{b2}\right)}\right)+\mu_{q\left(\frac{1}{a_{b2}}\right)}\nu_{b2}\mu_{q\left(\frac{1}{\sigma_{2}^{2}}\right)}-0.5\left(\nu_{e}+n\right)\log\left(\left|B_{q\left(\sigma_{e}^{2}\right)}\right|\right)-0.5\left(\nu_{e}+1\right)\log\left(B_{q\left(a_{e}\right)}\right)+\mu_{q\left(\frac{1}{a_{e}}\right)}\nu_{e}\mu_{q\left(\frac{1}{\sigma_{e}^{2}}\right).}$$ (8)

To obtain (8), several substitutions were made for simplification; it is valid only for the VBME model proposed. In this algorithm, the updates of the parameters should be as given in the order proposed for the algorithm.

### Model implementation

Recall that the hyperparameters of the prior distributions are values given by the user in the Bayesian paradigm. In this case, the hyperparameters used for the BME model given in the previous section were: $\beta_{\beta }=0_{I},$ $\Sigma_{0}=I_{I},$ $v_{\beta }=v_{b_{1}}=vb_{2}=2,$ and $A_{\beta }=A_{b_{1}}=A_{b_{2}}=A_{2}=10,000.$ All these hyperparameters were chosen to lead weakly informative priors. The implementation of both models (BME and VBME) was done in the R statistical software (R Core Team 2016). The full conditional and posterior distributions given in Appendix A were used for the BME and VBME models, respectively. Also, for the VBME model, an R code is given in Appendix D. The BME model was implemented with the MCMC method with 40,000 iterations with a burn-in of 20,000 and a thinning of five; therefore, 4000 samples were used for inference.

### Assessing prediction accuracy

Prediction accuracy was assessed using 20 training (trn)-testing (tst) random partitions; we used this approach because one can obtain as many partitions as one needs with a replicated trn-tst design. The implemented cross-validation (CV1) mimics a situation where lines were evaluated in some environments for the trait of interest, but some lines are missing from all the other environments. We assigned 80% of the observations to the trn set, and the remaining 20% to the tst set. Of the variety of methods for comparing the predictive posterior distribution to the observed data (generally termed “posterior predictive checks”), we used the Pearson correlation. Models with higher correlation values indicate better predictions. Under the BME, the predicted observations were calculated as $\hat{Y}=X\hat{\beta }+Z_{1}{\hat{b}}_{1},+Z_{2}{\hat{b}}_{2}$ where $\hat{\beta },{\hat{b}}_{1}\;\text{and}\;{\hat{b}}_{2},$ are estimates of β, $b_{1},$ and $b_{2},$ these estimates correspond to the posterior mean of s collected Gibbs samplers to estimate the predicted values after discarding those used for the burning period, while under the VBME, $\hat{Y}=X\mu_{q}\left(\beta \right)+Z_{1}\mu_{q\left(b_{1}\right)}+Z_{2}\mu_{q\left(b_{2}\right)},$ as described in the algorithm for implementing the VBME.

### Experimental data sets

Here, we present the information on the data sets used for implementing the proposed models. A total of eight data sets were used (three maize and five wheat).

#### Maize data sets 1–3:

A total of 309 doubled haploid maize lines was phenotyped and genotyped; this is part of the data set used by Crossa *et al.* (2013) and Montesinos-López *et al.* (2016), which comprised a total of 504 doubled haploid lines derived by crossing and backcrossing eight inbred lines to form several full-sib families. Traits available in this data set include grain yield (Maize_GY), anthesis-silking interval (Maize_ASI), and plant height (Maize_PH); each of these traits was evaluated in three optimum rainfed environments (Env1, Env2, and Env3). The experimental field design used in each of the three environments was an alpha lattice incomplete block design with two replicates. Data were preadjusted using estimates of block and environmental effects derived from a linear model that accounted for the incomplete block design within environment, and for environmental effects.

The genomic data were obtained through genotyping-by-sequencing (GBS) for each maize chromosome; the number of markers after initial filtering and the number of markers after imputation were summarized in Crossa *et al.* (2013). Filtering was first done by removing markers that had >80% of the maize lines with missing values, and then markers with minor allele frequency ≤0.05 were deleted. The total numbers of GBS data were 681,257 single nucleotide polymorphisms (SNPs), and, after filtering for missing values and minor allele frequency, 158,281 SNPs were used for the analyses. About 20% of cells were missing in the filtered GBS information used for prediction; these missing values were replaced by their expected values before doing the prediction.

#### Wheat data sets 4–5:

The first two wheat data sets came from a total of 250 wheat lines that were extracted from a large set of 39 yield trials grown during the 2013–2014 crop season in Ciudad Obregon, Sonora, Mexico (Rutkoski *et al.* 2016). The trials were sown in mid-November and grown on beds with five and two irrigations plus drip irrigation. Days to heading (Wheat_DTHD) were recorded as the number of days from germination until 50% of spikes had emerged in each plot, in the first replicate of each trial, while plant height (Wheat_PTHT) was recorded in centimeters.

For the first two wheat data sets, GBS was used for genome-wide genotyping. Single nucleotide polymorphisms were called across all lines using the TASSEL GBS pipeline anchored to the genome assembly of Chinese Spring. Single nucleotide polymorphisms were extracted and markers were filtered so that percent missing data did not exceed 80 and 20%, respectively. Individuals with >80% missing marker data were removed, and markers were recorded as −1, 0 and 1, which indicate homozygous for the minor allele, heterozygous, and homozygous for the major allele, respectively. Next, markers with <0.01 minor allele frequency were removed, and missing data were imputed with the marker mean. A total of 12,083 markers remained after marker editing.

#### Wheat data set 6:

This data set, from CIMMYT’s Global Wheat Program, was used by Crossa *et al.* (2010) and Cuevas *et al.* (2016a,b) and includes 599 wheat lines derived from 25 yr (1979–2005) of Elite Spring Wheat Yield Trials (ESWYT). The environments represented in these trials are four agroclimatic regions (mega-environments). The phenotypic trait considered here was grain yield (Wheat_Yield1) of the 599 wheat lines evaluated in each of the four mega-environments. The 599 wheat lines were genotyped using 1447 Diversity Array Technology (DArT) markers generated by Triticarte Pty. Ltd. (Canberra, Australia; http://www.triticarte.com.au). Markers with a minor allele frequency (MAF) (0.05) were removed, and missing genotypes were imputed using samples from the marginal distribution of marker genotypes. The number of DArT markers after edition was 1279.

#### Wheat data sets 7–8:

These two data sets were described and used by Lopez-Cruz *et al.* (2015) and Cuevas *et al.* (2016b) for proposing a marker×environment interaction model. The phenotypic data consisted of adjusted grain yield (tons/hectare) records collected during three evaluation cycles of different inbred lines evaluated in different environments, and denoted as Wheat_Yield2 and Wheat_Yield3. The environments were three irrigation regimes (moderate drought stress, optimal irrigation, and drought stress), two planting systems (bed and flat planting), and two different planting dates (normal and late). All trials were planted using a lattice design with three replicates in each environment at CIMMYT’s main wheat breeding station in Cd. Obregon, México. The phenotype used in the analysis was the Best Linear Unbiased Estimate (BLUE) of grain yield obtained from a linear model applied to the alpha-lattice design of each cycle-environment combination. The trait Wheat_Yield2 had 693 wheat lines evaluated in four environments, and the trait Wheat_Yield3 included 670 wheat lines evaluated in four environments. Genotypes were derived using GBS technology, and markers with a MAF of 0.05 were removed. All markers had a high incidence of uncalled genotypes, so we applied thresholds for incidence of missing values, and focused on maintaining relatively large and similar numbers of markers per data set. After editing the missing markers, we had a total of 15,744 GBS markers for analyzing traits Wheat_Yield2 and Wheat_Yield3.

### Data availability

Phenotypic and genotypic data on the eight data sets included in this study can be downloaded from http://hdl.handle.net/11529/10907.

## Results

The results of this research are given in two sections. The first section compares the parameter estimates and implementation time under both models (BME and VBME). The second section compares the prediction accuracy of both models in each of the eight data sets under study.

### Comparing parameter estimates and the implementation time of models BME and VBME

Table 1 gives the parameter estimates under both models (BME and VBME) of the ***β*** coefficients, variance components ($\sigma_{1}^{2},$ $\sigma_{2}^{2}$ and $\sigma_{e}^{2}$), Pearson correlation between the observed and predicted phenotypes of the full data sets, and the time in minutes required to implement each model on the data sets under study. In general, the beta coefficient estimates obtained under the BME and VBME models are very similar, but there are substantial differences in the estimates of variance components, given that a little more than half of them were lower under the VBME model as compared to those estimates obtained under the BME. Also, in seven out of eight data sets, the Pearson correlation between observed and predicted genotypes was larger under the BME, while in seven out of eight data sets, the implementation time was ∼10 times lower under the VBME than under BME.

**Table 1:** Parameter estimates, implementation time and Pearson correlation between the observed and predicted values for the complete data sets under the BME and VBME models

Model	Data Set	Parameter	$\beta_{1}$	$\beta_{2}$	$\beta_{3}$	$\beta_{4}$	$\sigma_{1}^{2}$	$\sigma_{2}^{2}$	$\sigma_{1}^{2}$	Correlation	Time
BME	Maize_GY	Mean	6.589	4.910	6.167	—	2.127	1.970	0.428	0.879	48.9
		SD	0.317	0.280	0.294	—	0.467	0.409	0.026	—	—
	Maize_ASI	Mean	1.837	1.168	2.222	—	2.909	1.290	0.436	0.855	50.88
		SD	0.361	0.340	0.352	—	0.535	0.326	0.026	—	—
	Maize_PH	Mean	2.330	2.021	2.343	—	0.007	0.002	0.020	0.745	49.68
		SD	0.044	0.043	0.043	—	0.009	0.003	0.002	—	—
	Wheat_DTHD	Mean	−3.155	−4.018	−0.298	—	26.381	8.015	0.403	0.999	64.62
		SD	0.270	0.268	0.276	—	2.656	0.545	0.029	—	—
	Wheat_PTHT	Mean	−4.652	−7.402	−0.776	—	8.864	25.702	0.403	0.999	31.14
		SD	0.269	0.262	0.267	—	1.725	1.687	0.029	—	—
	Wheat_Yield1	Mean	0.000	0.000	0.000	0.000	0.217	0.338	0.555	0.801	26.34
		SD	0.536	0.536	0.538	0.536	0.049	0.042	0.023	—	—
	Wheat_Yield2	Mean	0.170	0.202	0.073	0.082	0.295	0.230	0.486	0.816	33.36
		SD	0.476	0.475	0.476	0.477	0.039	0.027	0.018	—	—
	Wheat_Yield3	Mean	0.053	−0.072	−0.107	−0.079	0.402	0.244	0.520	0.810	31.98
		SD	0.587	0.585	0.584	0.586	0.055	0.032	0.021	—	—
VBME	Maize_GY	Mean	6.391	5.017	6.113	—	1.485	0.301	0.576	0.772	5.64
		SD	0.043	0.043	0.043	—	0.121	0.013	0.027	—	—
	Maize_ASI	Mean	1.858	1.143	2.364	—	1.825	0.282	0.503	0.781	5.64
		SD	0.040	0.040	0.040	—	0.148	0.013	0.023	—	—
	Maize_PH	Mean	2.337	2.051	2.333	—	0.011	0.011	0.014	0.859	3.18
		SD	0.007	0.007	0.007	—	0.0009	0.0005	0.0006	—	
	Wheat_DTHD	Mean	−3.243	−4.080	−0.321	—	25.063	0.451	6.161	0.943	1.2
		SD	0.161	0.161	0.161	—	2.274	0.003	0.337	—	—
	Wheat_PTHT	Mean	−4.594	−7.459	−0.611	—	6.796	2.937	17.009	0.846	1.2
		SD	0.260	0.260	0.260	—	0.627	0.132	0.893	—	—
	Wheat_Yield1	Mean	0.000	0.000	0.000	0.000	0.268	0.263	0.604	0.784	15.12
		SD	0.044	0.043	0.044	0.067	0.013	0.002	0.029	—	—
	Wheat_Yield2	Mean	0.172	0.193	0.091	0.091	0.250	0.118	0.542	0.785	38.94
		SD	0.027	0.027	0.027	0.027	0.014	0.003	0.015	—	—
	Wheat_Yield3	Mean	0.045	−0.062	−0.092	−0.075	0.417	0.190	0.561	0.797	25.86
		SD	0.028	0.028	0.028	0.028	0.023	0.005	0.015	—	—

The implementation time is given in minutes. Mean and SD under the BME were obtained as the Mean and SD posteriors, and, under the VBME, the Mean and SD were obtained as the mean and SD of the variational posterior.

Table 2 shows details of the comparison between the proposed VBME model *vs.* the BME model. The smallest difference between the beta coefficients was observed in $\beta_{1}$ for data set Wheat_Yield1 (1%), while the largest difference was observed in $\beta_{3}$ for data set Wheat_Yield2 (25.3%); the estimates of the BME model were lower. With regard to the variance components, Table 2 shows that the smallest difference was observed in $\sigma_{1}^{2}$ for Wheat_Yield3 (3.8%), while the largest difference was also in $\sigma_{1}^{2}$ for data set Maize_PH, where the estimated variance component of the VBME was 5.68 times higher than the estimated variance component of the BME model.

**Table 2:** Relative comparison using the full data sets between the BME and VBME calculated as parameter estimate under BME minus parameter estimate under VBME divided by parameter estimate under BME

Data Set	$\beta_{1}$	$\beta_{2}$	$\beta_{3}$	$\beta_{4}$	$\sigma_{1}^{2}$	$\sigma_{2}^{2}$	$\sigma_{e}^{2}$	Corr	Time
Maize_GY	0.030	−0.022	0.009	—	0.302	0.847	−0.346	0.122	0.885
Maize_ASI	−0.012	0.022	−0.064	—	0.372	0.781	−0.152	0.087	0.890
Maize_PH	−0.003	−0.015	0.004	—	**−0.568**	−5.289	0.330	−0.153	0.936
Wheat_DTHD	−0.028	−0.015	−0.075	—	0.050	0.944	−14.272	0.056	**0.982**
Wheat_PTHT	0.012	−0.008	0.213	—	0.233	0.886	−41.243	**0.154**	0.961
Wheat_Yield1	**−0.001**	−0.009	−0.068	−0.029	−0.235	0.222	−0.087	0.022	0.426
Wheat_Yield2	−0.010	0.047	**−0.253**	−0.110	0.153	0.488	−0.115	0.038	**−0.169**
Wheat_Yield3	0.160	0.148	0.144	0.055	**−0.038**	0.221	−0.079	**0.016**	0.190

The smallest and largest differences in *β* coefficients, variance components, Pearson correlation and implementation time are in boldface.

In terms of the Pearson correlation, in seven out of eight data sets, the best correlations were observed in the BME model; the minimum difference (1.6%) was observed in data set Wheat_Yield3, while the maximum difference (15.4%) was observed in Wheat_PTHT. Finally, in terms of implementation time (in minutes), in seven out of eight data sets the VBME model was faster than the BME model, with a minimum difference of 16.9%, and a maximum difference of 98.2%. The computer used to implement both models for each of the eight data sets (reported in Table 1 and Table 2) was a Windows operating system with processor Intel(R) Core(TM) i7-4610M CPU @ 3.0 GHz under a 64-bit operating system.

### Comparing the prediction ability of models BME and VBME

Table 3 presents prediction accuracies for both models resulting from the 20 trn-tst random partitions implemented for each of the eight real data sets under study. Table 3 gives the prediction accuracies for each environment in each data set. In general, the prediction accuracies of the BME are slightly higher than those of the VBME model. However, it is interesting to point out that, even under the VBME, the prediction accuracies are comparable to those found under the BME.

**Table 3:** Prediction accuracy of the BME and VBME models resulting from the 20 trn-tst random partitions implemented

		Env1		Env2		Env3		Env4	
Model	Data set	APC	SE	APC	SE	APC	SE	APC	SE
BME	Maize_GY	0.319	0.041	0.378	0.032	0.362	0.040	—	—
	Maize_ASI	0.466	0.027	0.380	0.045	0.285	0.048	—	—
	Maize_PH	0.391	0.024	0.352	0.029	0.585	0.024	—	—
	Wheat_DTHD	0.905	0.011	0.835	0.012	0.897	0.008	—	—
	Wheat_PTHT	0.523	0.020	0.347	0.027	0.499	0.026	—	—
	Wheat_Yield1	0.191	0.013	0.644	0.013	0.593	0.007	0.556	0.000
	Wheat_Yield2	0.690	0.015	0.785	0.010	0.617	0.021	0.637	0.020
	Wheat_Yield3	0.627	0.030	0.711	0.019	0.669	0.024	0.719	0.022
VBME	Maize_GY	0.341	0.025	0.383	0.027	0.283	0.021	—	—
	Maize_ASI	0.473	0.022	0.427	0.027	0.369	0.025	—	—
	Maize_PH	0.313	0.032	0.380	0.029	0.389	0.027	—	—
	Wheat_DTHD	0.920	0.010	0.860	0.012	0.908	0.008	—	—
	Wheat_PTHT	0.425	0.034	0.439	0.040	0.398	0.035	—	—
	Wheat_Yield1	0.455	0.015	0.527	0.012	0.434	0.010	0.262	0.006
	Wheat_Yield2	0.661	0.007	0.706	0.007	0.557	0.008	0.598	0.003
	Wheat_Yield3	0.432	0.012	0.490	0.011	0.480	0.011	0.514	0.010

APC, average of Pearson’s correlation; SE, standard error.

Table 4 shows that in five out of eight data sets the predictions under the BME model were the best. Also, the lowest difference in the five data sets in which the BME model was the best was 4.2% in favor of the BME in Env1 in data set Wheat_Yield2, while the largest difference was 52.9% in favor of the BME in Env4 in data set Wheat-Yield1. For the three data sets in which the VBME was the best, the smallest difference was 1.2% in Env2 in data set Wheat_DTHD, and the largest difference was 138.1% in Env1 in data set Wheat_Yield1.

**Table 4:** Relative comparison of the prediction accuracies of the BME and VBME models calculated as APC under BME minus APC VBME divided by the APC under BME

Data set	Env1	Env2	Env3	Env4
Maize_GY	−0.069	−0.015	0.217	—
Maize_ASI	−0.015	−0.121	−0.298	—
Maize_PH	0.198	−0.079	0.334	—
Wheat_DTHD	−0.017	−0.030	**−0.012**	—
Wheat_PTHT	0.187	−0.266	0.203	—
Wheat_Yield1	**−1.381**	0.182	0.268	**0.529**
Wheat_Yield2	**0.042**	0.101	0.097	0.061
Wheat_Yield3	0.311	0.311	0.282	0.285
Maize_GY	0	0	1	—
Maize_ASI	0	0	0	—
Maize_PH	1	0	1	—
Wheat_DTHD	0	0	0	—
Wheat_PTHT	1	0	1	—
Wheat_Yield1	0	1	1	1
Wheat_Yield2	1	1	1	1
Wheat_Yield3	1	1	1	1

The smallest and largest differences in terms of prediction accuracy for both models are in boldface. In the bottom half of this table, 1 means that the best model was BME and 0 means that the best model was VBME in terms of prediction accuracy.

## Discussion

We proposed and implemented the variational Bayes version of the BME model. We found that the *β* coefficients were estimated in a similar way in both models (BME and VBME), but the variance components were considerably underestimated in the VBME model. However, in terms of prediction accuracy, the BME was a little better than the VBME model. The VBME model is a quick and a good approximation to the BME model. These results were expected, since, under MCMC methods, we estimate the parameters using the mean posterior from samples of the target distribution (posterior distribution), while, in the second approach for estimating the parameters, we also use the mean, but from the approximated target distribution, here obtained under the VBM and called variational posterior distribution. The above is documented in some papers that reported that the VBM showed less accuracy than its MCMC counterpart. This is due to the fact that variational Bayes methods approximate the target posterior distribution by a factorized distribution that has minimum KL distance to the target posterior. The factorized distribution we used was the mean field variational family restriction, which makes strong independent assumptions to achieve great flexibility and scalable optimization at the price of low accuracy and underestimation of posterior variances (Blei *et al.* 2016). In contrast, MCMC methods such as the BME model have a high computational cost with the advantage that they generate samples directly from the target posterior distribution that are guaranteed to converge asymptotically to the true posterior distribution. The new proposed VBME model for univariate phenotypes with G×E interaction is a step ahead of the models proposed until now, in the context of genomic-enabled prediction models proposed by other authors and described in the *Introduction*. In this paper, we propose a genomic VBM model using a half-*t* priors distribution, and give clear theoretical details of the derivation of the full conditional and posterior distributions, and account for G×E interaction.

Advances in genetic engineering allow collecting and storing millions of markers (SNPs, etc.) from plant and animal genomes, and many breeding programs are interested in improving many correlated traits simultaneously. For these reasons, in genomic selection there is a great need to use genome-enabled prediction models that take into account all available markers, traits, and environments under study to improve the prediction of genotypes early in time for multiple correlated traits. However, due to the large amount of input information for Bayesian methods (such as those used in the BME model), these statistical models and methods might become intractable, or impose a very high computational cost in terms of time. For these reasons, and because it offers a significant improvement in computational speed at the cost of a small loss in prediction accuracy, we believe that the proposed VBME model is an attractive alternative to the BME model.

### Advantages and disadvantages of the variational model (VBME)

As already mentioned, the variational model (VBME) was proposed due to the need to improve the speed of the BME. For this reason, we propose the iterative algorithm given in the section *Algorithm for implementing the VBME*, which approximates the BME model in a reasonable way. Results show that the VBME method: (1) is sometimes more flexible than its Bayesian counterpart; (2) offers a reasonable approximation to the posterior distributions of all model parameters; (3) most of the time is much less demanding in computational time than the BME built using MCMC techniques; and (4) maintains most of the advantages of Bayesian inference. However, the VBME has some limitations: (1) the approximation cannot become more accurate by increasing computational time; (2) part of the VBME’s advantage in computing time is because the model is simple; however, this algorithm needs to be adapted to cases that are different from the one used here; (3) the VBME, like all variational Bayes methods, does not guarantee that it will converge to a global maximum; (4) the convergence time depends strongly on the level of tolerance desired and the nature of the data; and (5) it has been documented, and was observed here, that variational models underestimate some covariance parameters. However, the time reduction depends strongly on the tolerance level: the lower the value, the longer the convergence time. For this reason, although our results show a significant reduction in time, this gain can be reduced substantially if the chosen tolerance level is smaller. Our empirical results when implementing the VBME in eight real data sets show that a tolerance level of 0.00001 is enough, because when we increased it to 1E−8, no significant gain in prediction accuracy or accuracy in parameter estimates was observed. Also, since in developing our VBME, we used the mean field variational family restriction that approximates q(θ) by the product of q latent independent variables to gain tractability, this may underestimate the variability of parameter estimates when there is a considerable degree of dependence. Another important disadvantage of the VBM is that deriving the set of equations (variational posterior and lower bound) used to iteratively update the parameters often requires a large amount of algebra compared with deriving the comparable Gibbs sampling equations. This is evident in Appendix A for our proposed VBME model, which, despite being a simple model, required a lot of algebra and notation to obtain the variational posterior distribution and the corresponding lower bound. This amount of algebra needs to be done each time for every specific model.

To take advantage of the proposed model, we provide some guidance on when to use the VBME or the BME. The BME (an MCMC method) tends to be more computationally intense than the VBME (a variational method) but guarantees that exact samples from the target density are (asymptotically) produced (Robert 2004). While the VBME does not guarantee exact samples, only approximate ones (that is, it can only find a density close to the target), most of the time it is faster than MCMC methods. Since the VBME is based on optimization, it can more easily take advantages of stochastic optimization and distributed optimization. For these reasons, the VBME is expected to be better suited to larger data sets than the BME and for scenarios where we want to quickly explore many models. Also, Blei *et al.* (2016) point out that the VBME is preferred when the posterior of a mixture model admits multiple modes, since MCMC techniques are not an option for this scenario, even with small data sets.

Finally, we believe that the VBME method, as proposed here, is a useful addition to the existing arsenal of genome-enabled prediction models; it is also an alternative to the BME model, since it considerably reduces implementation time. Additionally, this research opens a new branch of methodologies for fitting genomic models to genomic data that need novel methods to take advantage of the huge amount of data available nowadays. For these reasons, we agree with Ormerod and Wand (2010), who pointed out that the usefulness of variational approximations increases as the size of the problem increases, and Monte Carlo methods such as MCMC start to become untenable. We also believe that our research contributes significantly to researchers’ understanding of the VBM, because, in developing the proposed VBME, we provided all the details of the derivation of the variational posterior distributions needed to derive the q-specific evidence lower bound for building the algorithm, and the R scripts for implementing the proposed VBME model. This information gives the scientist the basic elements needed to develop new models under the variational Bayes approach. It is also important to point out that we provide all the details for deriving the full conditionals needed for implementing the BME model since the proposed model uses Half-t priors that produce noninformative variance components (Huang and Wand 2013).

### Conclusions

In this paper, we propose a new alternative to the BME via variational Bayes, which we called the VBME model. The proposed method underestimated the variance components and similar estimates of the beta coefficients were found; in terms of prediction accuracy, the VBME had lower prediction accuracies than the BME. However, according to our results, the loss in prediction accuracy is compensated for by the significant gain in implementation time, since the VBME is much faster than the BME. However, it is important to point out that if a very low tolerance value is selected, the gain in terms of implementation speed will vanish. For this reason, to take advantage of the proposed VBME, a reasonable tolerance value should be chosen that guarantees the quality of the parameter estimates and predictions, as well as a significant reduction in implementation time.

We also believe that the proposed model should be tested on many real data sets to obtain more empirical evidence of its performance; this evidence would allow us to extend the proposed variational Bayes model to take into account nonnormal responses (binary, Poisson, negative binomial, etc.), for multiple traits and multiple-environment models (Montesinos-López *et al.* 2016), which would enable scientists to make a more precise selection of candidate genotypes at early crop stages. Also, models that avoid the product density restriction can be proposed. Finally, the proposed VBME model is a useful addition to the existing arsenal of genome-enabled prediction models, and an excellent alternative to the BME model, since it produces competitive predictions at a lower computational cost.

## Appendix A

### Derivation of full conditional and variational posterior distributions for the BME and VBME models

#### Full conditional for β

$$P\left(\beta |ELSE\right)\propto P\left(Y|\beta ,b_{1},b_{2},\sigma_{e}^{2}\right)P\left(\beta |\sigma_{\beta }^{2}\right)\propto \text{exp}\left\{-\frac{1}{2}{\left(Y-X\beta -Z_{1}b_{1}-Z_{2}b_{2}\right)}^{T}{\left(I_{n}\sigma_{e}^{2}\right)}^{-1}\left(Y-X\beta -Z_{1}b_{1}-Z_{2}b_{2}\right)\right\}\times \text{exp}\left\{-\frac{1}{2}\left({\left[\beta -\beta_{\beta }\right]}^{T}\Sigma_{\beta }^{-1}\sigma_{\beta }^{-2}\left[\beta -\beta_{\beta }\right]\right)\right\}\propto \text{exp}\left\{-\frac{1}{2}{\left(\beta -\tilde{\beta }\right)}^{T}{\tilde{\Sigma }}_{\beta }^{-1}\left(\beta -\tilde{\beta }\right)\right\}\propto N\left(\tilde{\beta },{\tilde{\Sigma }}_{\beta }\right)$$

where:

$${\tilde{\Sigma }}_{\beta }={\left(\Sigma_{\beta }^{-1}\sigma_{\beta }^{-2}+X^{T}{\left(I_{n}\sigma_{e}^{2}\right)}^{-1}X\right)}^{-1};\tilde{\beta }={\tilde{\Sigma }}_{\beta }\left(\Sigma_{\beta }^{-1}\beta_{\beta }\sigma_{\beta }^{-2}+X^{T}{\left(I_{n}\sigma_{e}^{2}\right)}^{-1}Y-X^{T}{\left(I_{n}\sigma_{e}^{2}\right)}^{-1}Z_{1}b_{1}-X^{T}{\left(I_{n}\sigma_{e}^{2}\right)}^{-1}Z_{2}b_{2}\right)$$

#### Variational posterior for β

$$q\left(\beta |ELSE\right)=\text{exp}\left\{E_{-\beta }\left[\text{log}P\left(\beta |ELSE\right)\right]\right\}\propto \text{exp}\left(-\frac{1}{2}\left\{E_{-\beta }\left[\beta^{T}\left(\Sigma_{\beta }^{-1}\sigma_{\beta }^{-2}+X^{T}{\left(I_{n}\sigma_{e}^{2}\right)}^{-1}X\right)\beta -2{\left(\Sigma_{\beta }^{-1}\beta_{\beta }\sigma_{\beta }^{-2}+X^{T}{\left(I_{n}\sigma_{e}^{2}\right)}^{-1}Y-X^{T}{\left(I_{n}\sigma_{e}^{2}\right)}^{-1}Z_{1}b_{1}-X^{T}{\left(I_{n}\sigma_{e}^{2}\right)}^{-1}Z_{2}b_{2}\right)}^{T}\beta \right]\right\}\right)\propto \text{exp}\left(-\frac{1}{2}\left[{\left(\beta -\mu_{q\left(\beta \right)}\right)}^{T}\Sigma_{q\left(\beta \right)}^{-1}\left(\beta -\mu_{q\left(\beta \right)}\right)\right]\right)\propto N\left(\mu_{q\left(\beta \right)},\Sigma_{q\left(\beta \right)}\right)$$ (A.11)

where

$$\Sigma_{q\left(\beta \right)}={\left(\Sigma_{\beta }^{-1}\mu_{q\left(1/\sigma_{\beta }^{2}\right)}+X^{T}\left(I_{n}\mu_{q\left(\sigma_{e}^{-2}\right)}\right)X\right)}^{-1},\mu_{q\left(\beta \right)}=\Sigma_{q\left(\beta \right)}\left(\Sigma_{\beta }^{-1}\beta_{\beta }\mu_{q\left(1/\sigma_{\beta }^{2}\right)}+X^{T}\left[I_{n}\mu_{q\left(\sigma_{e}^{-2}\right)}\right]\left[Y-\sum_{h=1}^{2}Z_{h}\mu_{q\left(b_{h}\right)}\right]\right)$$

#### Full conditional for $\sigma_{\beta }^{2}$

$$P\left(\sigma_{\beta }^{2}|ELSE\right)\propto P\left(\beta |\sigma_{\beta }^{2}\right)P\left(\sigma_{\beta }^{2}|a_{\beta }\right)\propto {\left|\Sigma_{\beta }\right|}^{-\frac{1}{2}}{\left(\sigma_{\beta }^{2}\right)}^{-\frac{I}{2}}\text{exp}\left\{-\frac{1}{2}\left(\beta^{T}\Sigma_{\beta }^{-1}\sigma_{\beta }^{-2}\beta \right)\right\}{\left(\sigma_{\beta }^{2}\right)}^{-\frac{V_{\beta }}{2}-1}{\left(\frac{\nu_{\beta }}{a_{\beta }}\right)}^{\frac{\nu_{\beta }}{2}}\text{exp}\left\{-\frac{\nu_{\beta }}{a_{\beta }}\frac{1}{\sigma_{\beta }^{2}}\right\}\propto {\left(\sigma_{\beta }^{2}\right)}^{-\frac{\nu_{\beta }+I}{2}-1}\text{exp}\left\{-\left(\frac{{\left[\beta -\beta_{\beta }\right]}^{T}\Sigma_{\beta }^{-1}\left[\beta -\beta_{\beta }\right]}{2}+\frac{\nu_{\beta }}{a_{\beta }}\right)\left(\frac{1}{\sigma_{\beta }^{2}}\right)\right\}\propto IG\left(\frac{\nu_{\beta }+I}{2},\frac{{\left[\beta -\beta_{\beta }\right]}^{T}\Sigma_{\beta }^{-1}\left[\beta -\beta_{\beta }\right]}{2}+\frac{\nu_{\beta }}{a_{\beta }}\right)$$

#### Variational posterior for $\sigma_{\beta }^{2}$

$$q\left(\sigma_{\beta }^{2}|ELSE\right)\propto \text{exp}\left\{E_{-\sigma_{\beta }^{2}}\left[\log P\left(\sigma_{\beta }^{2}|ELSE\right)\right]\right\}\propto \frac{1}{{\left(\sigma_{\beta }^{2}\right)}^{\frac{\nu_{\beta }+1+I+1}{2}}}\text{exp}\left(-\frac{1}{\sigma_{\beta }^{2}}\left\{E_{-\sigma_{\beta }^{2}}\left(\frac{{\left[\beta -\beta_{\beta }\right]}^{T}\Sigma_{\beta }^{-1}\left[\beta -\beta_{\beta }\right]}{2}+\nu_{\beta }/a_{\beta }\right)\right\}\right)\propto \frac{1}{{\left(\sigma_{\beta }^{2}\right)}^{\frac{\nu_{\beta }+1+I+1}{2}}}\text{exp}\left(-\frac{1}{\sigma_{\beta }^{2}}\left\{\frac{{\left[\mu_{q\left(\beta \right)}-\beta_{\beta }\right]}^{T}\Sigma_{\beta }^{-1}\left[\mu_{q\left(\beta \right)}-\beta_{\beta }\right]+tr\left(\Sigma_{\beta }^{-1}\Sigma_{q\left(\beta \right)}\right)}{2}+\nu_{\beta }\mu_{q\left(1/a_{\beta }\right)}\right\}\right)\propto IG\left(k_{q\left(\sigma_{\beta }^{2}\right)}=\frac{\nu_{\beta }+I}{2},B_{q\left(\sigma_{\beta }^{2}\right)}=\frac{{\left[\mu_{q\left(\beta \right)}-\beta_{\beta }\right]}^{T}\Sigma_{\beta }^{-1}\left[\mu_{q\left(\beta \right)}-\beta_{\beta }\right]+tr\left(\Sigma_{\beta }^{-1}\Sigma_{q\left(\beta \right)}\right)}{2}+\nu_{\beta }\mu_{q\left(1/a_{\beta }\right)}\right)$$

#### Full conditional for $a_{\beta }$

$$P\left(a_{\beta }|ELSE\right)\propto P\left(\sigma_{\beta }^{2}|a_{\beta }\right)P\left(a_{\beta }\right)\propto {\left(\sigma_{\beta }^{2}\right)}^{-\frac{\nu_{\beta }}{2}-1}{\left(\frac{\nu_{\beta }}{a_{\beta }}\right)}^{\frac{\nu_{\beta }}{2}}\text{exp}\left\{-\frac{\nu_{\beta }}{a_{\beta }}\frac{1}{\sigma_{\beta }^{2}}\right\}{\left(a_{\beta }\right)}^{-\frac{1}{2}-1}{\left(\frac{1}{A_{\beta }^{2}}\right)}^{\frac{1}{2}}\text{exp}\left\{-\frac{1}{A_{\beta }^{2}}\frac{1}{a_{\beta }}\right\}\propto {\left(a_{\beta }\right)}^{-\frac{\nu_{\beta }+1}{2}-1}\text{exp}\left\{-\left(\frac{\nu_{\beta }}{\sigma_{\beta }^{2}}+\frac{1}{A_{\beta }^{2}}\right)\left(\frac{1}{a_{\beta }}\right)\right\}\propto IG\left(\frac{\nu_{\beta }+1}{2},\frac{\nu_{\beta }}{\sigma_{\beta }^{2}}+\frac{1}{A_{\beta }^{2}}\right)$$

#### Variational posterior for $a_{\beta }$

$$q\left(a_{\beta }|ELSE\right)\propto \text{exp}\left\{E_{-a_{\beta }}\left[\text{logP}\left(a_{\beta }|ELSE\right)\right]\right\}\propto \frac{1}{{\left(a_{\beta }\right)}^{\frac{\nu_{\beta }+1}{2}+1}}\text{exp}\left(-\frac{1}{a_{\beta }}\left\{E_{-a_{\beta }}\left(\frac{1}{A_{\beta }^{2}}+\frac{\nu_{\beta }}{\sigma_{\beta }^{2}}\right)\right\}\right)\propto IG\left(k_{q\left(a_{\beta }\right)}=\frac{\nu_{\beta }+1}{2},B_{q\left(a_{\beta }\right)}=1/A_{\beta }^{2}+\nu_{\beta }\mu_{q\left(1/\sigma_{\beta }^{2}\right)}\right)$$

#### Full conditional for $b_{1}$

$$P\left(b_{1}|ELSE\right)\propto P\left(Y|\beta ,b_{1},b_{2},\sigma_{e}^{2}\right)P\left(b_{1}|\sigma_{b_{1}}^{2}\right)\propto \text{exp}\left\{-\frac{1}{2}{\left(Y-X\beta -Z_{1}b_{1}-Z_{2}b_{2}\right)}^{T}{\left(I_{n}\sigma_{e}^{2}\right)}^{-1}\left(Y-X\beta -Z_{1}b_{1}-Z_{2}b_{2}\right)\right\}\times \text{exp}\left\{-\frac{1}{2}\left(b_{1}^{T}G^{-1}\sigma_{b_{1}}^{-2}b_{1}\right)\right\}\propto \text{exp}\left\{-\frac{1}{2}{\left(b_{1}-\tilde{b_{1}}\right)}^{T}{\tilde{\Sigma }}_{b_{1}}^{-1}\left(b_{1}-\tilde{b_{1}}\right)\right\}\propto N\left(\tilde{b_{1}},{\tilde{\Sigma }}_{b_{1}}\right)$$

where

$${\tilde{\Sigma }}_{b_{1}}={\left(G^{-1}\sigma_{b_{1}}^{-2}+Z_{1}^{T}{\left(I_{n}\sigma_{e}^{2}\right)}^{-1}Z_{1}\right)}^{-1};\tilde{b_{1}}={\tilde{\Sigma }}_{b_{1}}\left(Z_{1}^{T}{\left(I_{n}\sigma_{e}^{2}\right)}^{-1}Y-Z_{1}^{T}{\left(I_{n}\sigma_{e}^{2}\right)}^{-1}X\beta -Z_{1}^{T}{\left(I_{n}\sigma_{e}^{2}\right)}^{-1}Z_{2}b_{2}\right)$$

#### Variational posterior for $b_{1}$

Defining $\eta^{1}=X\beta +Z_{2}b_{2}$, the variational posterior distribution of $b_{1}$ is given as

$$q\left(b_{1}|ELSE\right)\propto \text{exp}\left\{E_{-b_{1}}\left[\text{logP}\left(b_{1}|ELSE\right)\right]\right\}\propto \text{exp}\left\{-\frac{1}{2}E_{-b_{1}}\left[b_{1}^{T}\left(G^{-1}\sigma_{1}^{-2}+Z_{1}^{T}{\left(I_{n}\sigma_{e}^{2}\right)}^{-1}Z_{1}\right)b_{1}-2{\left(Z_{1}^{T}{\left(I_{n}\sigma_{e}^{2}\right)}^{-1}Y-Z_{1}^{T}{\left(I_{n}\sigma_{e}^{2}\right)}^{-1}\eta^{1}\right)}^{T}b_{1}\right]\right\}\propto \text{exp}\left\{-\frac{1}{2}{\left(b_{1}-\mu_{q\left(b_{1}\right)}\right)}^{T}\Sigma_{q\left(b_{1}\right)}^{-1}\left(b_{1}-\mu_{q\left(b_{1}\right)}\right)\right\}\propto N\left(\mu_{q\left(b_{1}\right)},\Sigma_{q\left(b_{1}\right)}\right)$$

where

$$\Sigma_{q\left(b_{1}\right)}={\left(G^{-1}\mu_{q\left(\sigma_{1}^{-2}\right)}+Z_{1}^{T}\left[I_{n}\mu_{q\left(\sigma_{e}^{-2}\right)}\right]Z_{1}\right)}^{-1}\;\text{and}\;\mu_{q\left(b_{1}\right)}=\Sigma_{q\left(b_{1}\right)}\left(Z_{1}^{T}\left[I_{n}\mu_{q\left(\sigma_{e}^{-2}\right)}\right]\left[Y-X\mu_{q\left(\beta \right)}-Z_{2}\mu_{q\left(b_{2}\right)}\right]\right)$$

#### Full conditional for $\sigma_{1}^{2}$

$$P\left(\sigma_{1}^{2}|ELSE\right)\propto P\left(b_{1}|\sigma_{1}^{2}\right)P\left(\sigma_{1}^{2}|a_{b_{1}}\right)\propto {\left|G\right|}^{-\frac{1}{2}}{\left(\sigma_{1}^{2}\right)}^{-\frac{J}{2}}\text{exp}\left\{-\frac{1}{2}\left(b_{1}^{T}G^{-1}\sigma_{1}^{-2}b_{1}\right)\right\}{\left(\sigma_{1}^{2}\right)}^{-\frac{\nu_{b_{1}}}{2}-1}{\left(\frac{\nu_{b_{1}}}{a_{b_{1}}}\right)}^{\frac{\nu_{b_{1}}}{2}}\text{exp}\left\{-\frac{\nu_{b_{1}}}{a_{b_{1}}}\frac{1}{\sigma_{b_{1}}^{2}}\right\}\propto {\left(\sigma_{1}^{2}\right)}^{-\frac{\nu_{b_{1}}+J}{2}-1}\text{exp}\left\{-\left(\frac{b_{1}^{T}G^{-1}b_{1}}{2}+\frac{\nu_{b_{1}}}{a_{b_{1}}}\right)\left(\frac{1}{\sigma_{b_{1}}^{2}}\right)\right\}\propto IG\left(\frac{\nu_{b_{1}}+J}{2},\frac{b_{1}^{T}G^{-1}b_{1}}{2}+\frac{\nu_{b_{1}}}{a_{b_{1}}}\right)$$

#### Variational posterior for $\sigma_{1}^{2}$

$$q\left(\sigma_{1}^{2}|ELSE\right)\propto \text{exp}\left\{E_{-\sigma_{b_{1}}^{2}}\left[\text{log}P\left(\sigma_{1}^{2}|ELSE\right)\right]\right\}\propto \frac{1}{{\left(\sigma_{1}^{2}\right)}^{\frac{\nu_{b_{1}}+1+J+1}{2}}}\text{exp}\left(-\frac{1}{\sigma_{1}^{2}}\left\{E_{-\sigma_{b_{1}}^{2}}\left(\frac{b_{1}^{T}G^{-1}b_{1}}{2}+\frac{\nu_{b_{1}}}{a_{b_{1}}}\right)\right\}\right)\propto \frac{1}{{\left(\sigma_{1}^{2}\right)}^{\frac{\nu_{b_{1}}+1+J+1}{2}}}\text{exp}\left(-\frac{1}{\sigma_{1}^{2}}\left\{\frac{\mu_{q\left(b_{1}\right)}^{T}G^{-1}\mu_{q\left(b_{1}\right)}+tr\left(G^{-1}\Sigma_{q\left(b_{1}\right)}\right)}{2}+\nu_{b_{1}}\mu_{q\left(1/a_{b_{1}}\right)}\right\}\right)\propto IG\left(k_{q\left(\sigma_{1}^{2}\right)}=\frac{\nu_{b_{1}}+J}{2},B_{q\left(\sigma_{1}^{2}\right)}=\frac{\mu_{q\left(b_{1}\right)}^{T}G^{-1}\mu_{q\left(b_{1}\right)}+tr\left(G^{-1}\Sigma_{q\left(b_{1}\right)}\right)}{2}+\nu_{b_{1}}\mu_{q\left(1/a_{b_{1}}\right)}\right)$$

#### Full conditional for $a_{b_{1}}$

$$P\left(a_{b_{1}}|ELSE\right)\propto P\left(\sigma_{1}^{2}|a_{b_{1}}\right)P\left(a_{b_{1}}\right)\propto {\left(\sigma_{1}^{2}\right)}^{-\frac{\nu_{b_{1}}}{2}-1}{\left(\frac{\nu_{b_{1}}}{a_{b_{1}}}\right)}^{\frac{\nu_{b_{1}}}{2}}\text{exp}\left\{-\frac{\nu_{b_{1}}}{a_{b_{1}}}\frac{1}{\sigma_{b_{1}}^{2}}\right\}{\left(a_{b_{1}}\right)}^{-\frac{1}{2}-1}{\left(\frac{1}{A_{b_{1}}^{2}}\right)}^{\frac{1}{2}}\text{exp}\left\{-\frac{1}{A_{b_{1}}^{2}}\frac{1}{a_{b_{1}}}\right\}\propto {\left(a_{b_{1}}\right)}^{-\frac{\nu_{b_{1}}+1}{2}-1}\text{exp}\left\{-\left(\frac{\nu_{b_{1}}}{\sigma_{1}^{2}}+\frac{1}{A_{b_{1}}^{2}}\right)\left(\frac{1}{a_{b_{1}}}\right)\right\}\propto IG\left(\frac{\nu_{b_{1}}+1}{2},\frac{\nu_{b_{1}}}{\sigma_{1}^{2}}+\frac{1}{A_{b_{1}}^{2}}\right)$$

#### Variational posterior for $a_{b_{1}}$

$$q\left(a_{b_{1}}|ELSE\right)\propto \text{exp}\left\{E_{-a_{b_{1}}}\left[\text{logP}\left(a_{b_{1}}|ELSE\right)\right]\right\}\propto \frac{1}{{\left(a_{b_{1}}\right)}^{\frac{\nu_{b_{1}}+1}{2}+1}}\text{exp}\left(-\frac{1}{a_{b_{1}}}\left\{E_{-a_{b_{1}}}\left(\frac{1}{A_{b_{1}}^{2}}+\frac{\nu_{b_{1}}}{\sigma_{1}^{2}}\right)\right\}\right)\propto IG\left(k_{q\left(a_{b_{1}}\right)}=\frac{\nu_{b_{1}}+1}{2},B_{q\left(a_{b_{1}}\right)}=1/A_{b_{1}}^{2}+\nu_{b_{1}}\mu_{q\left(1/\sigma_{1}^{2}\right)}\right)$$

#### Full conditional for $b_{2}$

$$P\left(b_{2}|ELSE\right)\propto P\left(Y|\beta ,b_{1},b_{2},\sigma_{e}^{2}\right)P\left(b_{2}|\sigma_{b_{2}}^{2}\right)\propto \text{exp}\left\{-\frac{1}{2}{\left(Y-X\beta -Z_{1}b_{1}-Z_{2}b_{2}\right)}^{T}{\left(I_{n}\sigma_{e}^{2}\right)}^{-1}\left(Y-X\beta -Z_{1}b_{1}-Z_{2}b_{2}\right)\right\}\times \text{exp}\left\{-\frac{1}{2}\left(b_{2}^{T}G_{2}^{-1}\sigma_{b_{2}}^{-2}b_{2}\right)\right\}\propto \text{exp}\left\{-\frac{1}{2}{\left(b_{2}-\tilde{b_{2}}\right)}^{T}{\tilde{\Sigma }}_{b_{2}}^{-1}\left(b_{2}-\tilde{b_{2}}\right)\right\}\propto N\left(\tilde{b_{2}},{\tilde{\Sigma }}_{b_{2}}\right)$$

where:

$${\tilde{\Sigma }}_{b_{2}}={\left(G_{2}^{-1}\sigma_{2}^{-2}+Z_{2}^{T}{\left(I_{n}\sigma_{e}^{2}\right)}^{-1}Z_{2}\right)}^{-1};\tilde{b_{2}}={\tilde{\Sigma }}_{b_{2}}\left(Z_{2}^{T}{\left(I_{n}\sigma_{e}^{2}\right)}^{-1}Y-Z_{2}^{T}{\left(I_{n}\sigma_{e}^{2}\right)}^{-1}X\beta -Z_{2}^{T}{\left(I_{n}\sigma_{e}^{2}\right)}^{-1}Z_{1}b_{1}\right)$$

#### Variational posterior for $b_{2}$

Defining $\eta^{2}=X\beta +Z_{1}b_{1},$ the variational posterior distribution of $b_{2}$ is given as

$$q\left(b_{2}|ELSE\right)\propto \text{exp}\left\{E_{-b_{2}}\left[\text{logP}\left(b_{2}|ELSE\right)\right]\right\}\propto \text{exp}\left\{-\frac{1}{2}{\left(b_{2}-\mu_{q\left(b_{2}\right)}\right)}^{T}\Sigma_{q\left(b_{2}\right)}^{-1}\left(b_{2}-\mu_{q\left(b_{2}\right)}\right)\right\}\propto N\left(\mu_{q\left(b_{2}\right)},\Sigma_{q\left(b_{2}\right)}\right)$$

where

$$\Sigma_{q\left(b_{2}\right)}={\left(G_{2}^{-1}\mu_{q\left(\sigma_{2}^{-2}\right)}+Z_{2}^{T}\left[I_{n}\mu_{q\left(\sigma_{e}^{-2}\right)}\right]Z_{2}\right)}^{-1}\;\text{and}\;\mu_{q\left(b_{2}\right)}=\Sigma_{q\left(b_{2}\right)}\left(Z_{2}^{T}\left[I_{n}\mu_{q\left(\sigma_{e}^{-2}\right)}\right]\left[Y-X\mu_{q\left(\beta \right)}-Z_{2}\mu_{q\left(b_{1}\right)}\right]\right)$$

#### Full conditional for $\sigma_{2}^{2}$

$$P\left(\sigma_{2}^{2}|ELSE\right)\propto P\left(b_{2}|\sigma_{2}^{2}\right)P\left(\sigma_{2}^{2}|a_{b_{2}}\right)\propto {\left|G_{2}\right|}^{-\frac{1}{2}}{\left(\sigma_{2}^{2}\right)}^{-\frac{IJ}{2}}\text{exp}\left\{-\frac{1}{2}\left(b_{2}^{T}G_{2}^{-1}\sigma_{2}^{-2}b_{2}\right)\right\}{\left(\sigma_{2}^{2}\right)}^{-\frac{\nu_{b_{2}}}{2}-1}{\left(\frac{\nu_{b_{2}}}{a_{b_{2}}}\right)}^{\frac{\nu_{b_{2}}}{2}}\text{exp}\left\{-\frac{\nu_{b_{2}}}{a_{b_{2}}}\frac{1}{\sigma_{b_{2}}^{2}}\right\}\propto {\left(\sigma_{2}^{2}\right)}^{-\frac{\nu_{b_{1}}+IJ}{2}-1}\text{exp}\left\{-\left(\frac{b_{2}^{T}G_{2}^{-1}b_{2}}{2}+\frac{\nu_{b_{2}}}{a_{b_{2}}}\right)\left(\frac{1}{\sigma_{b_{2}}^{2}}\right)\right\}\propto IG\left(\frac{\nu_{b_{2}}+IJ}{2},\frac{b_{2}^{T}G_{2}^{-1}b_{2}}{2}+\frac{\nu_{b_{2}}}{a_{b_{2}}}\right)$$

#### Variational posterior for $\sigma_{2}^{2}$

q(σ22|ELSE)∝exp{E−σ22[logP(σb22|ELSE)]}∝1(σ22)νb1+1+IJ+12exp(−1σ22{E−σb22(b2TG2−1b22+νb2ab2)})∝1(σ22)νb1+1+IJ+12exp(−1σ22{μq(b2)G2−1Tμq(b2)+tr(G2−1Σq(b2))2+νb2μq(1/ab2 )})∝IG(kq(σb22)=νb2+IJ2,Bq(σ22)=μq(b2)G2−1Tμq(b2)+tr(G2−1Σq(b2))2+νb2μq(1/ab2))

#### Full conditional for $a_{b_{2}}$

$$P\left(a_{b_{2}}|ELSE\right)\propto P\left(\sigma_{2}^{2}|a_{b_{2}}\right)P\left(a_{b_{2}}\right)\propto {\left(\sigma_{2}^{2}\right)}^{-\frac{\nu_{b_{2}}}{2}-1}{\left(\frac{\nu_{b_{2}}}{a_{b_{2}}}\right)}^{\frac{\nu_{b_{2}}}{2}}\text{exp}\left\{-\frac{\nu_{b_{2}}}{a_{b_{2}}}\frac{1}{\sigma_{b_{2}}^{2}}\right\}{\left(a_{b_{2}}\right)}^{-\frac{1}{2}-1}{\left(\frac{1}{A_{b_{2}}^{2}}\right)}^{\frac{1}{2}}\text{exp}\left\{-\frac{1}{A_{b_{2}}^{2}}\frac{1}{a_{b_{2}}}\right\}\propto {\left(a_{b_{2}}\right)}^{-\frac{\nu_{b_{2}}+1}{2}-1}\text{exp}\left\{-\left(\frac{\nu_{b_{2}}}{\sigma_{2}^{2}}+\frac{1}{A_{b_{2}}^{2}}\right)\left(\frac{1}{a_{b_{2}}}\right)\right\}\propto IG\left(\frac{\nu_{b_{2}}+1}{2},\frac{\nu_{b_{2}}}{\sigma_{2}^{2}}+\frac{1}{A_{b_{2}}^{2}}\right)$$

#### Variational posterior for $a_{b_{2}}$

$$q\left(a_{b_{2}}|ELSE\right)\propto \text{exp}\left\{E_{-a_{b_{2}}}\left[\text{logP}\left(a_{b_{2}}|ELSE\right)\right]\right\}\propto \frac{1}{{\left(a_{b_{2}}\right)}^{\frac{\nu_{b_{2}}+1}{2}+1}}\text{exp}\left(-\frac{1}{a_{b_{2}}}\left\{E_{-a_{b_{2}}}\left(\frac{1}{A_{b_{2}}^{2}}+\frac{\nu_{b_{2}}}{\sigma_{2}^{2}}\right)\right\}\right)\propto IG\left(k_{q\left(a_{b_{2}}\right)}=\frac{\nu_{b_{2}}+1}{2},B_{q\left(a_{b_{2}}\right)}=1/A_{b_{2}}^{2}+\nu_{b_{2}}\mu_{q\left(1/\sigma_{2}^{2}\right)}\right)$$

#### Full conditional for $\sigma_{e}^{2}$

$$P\left(\sigma_{\epsilon }^{2}|ELSE\right)\propto P\left(Y|\beta ,b_{1},b_{2},\sigma_{e}^{2}\right)P\left(\sigma_{e}^{2}|a_{e}\right)\propto {\left|I_{n}\right|}^{-\frac{1}{2}}{\left(\sigma_{e}^{2}\right)}^{-\frac{n}{2}}\text{exp}\left\{-\frac{1}{2}{\left(Y-X\beta -Z_{1}b_{1}-Z_{2}b_{2}\right)}^{T}{\left(I_{n}\sigma_{e}^{2}\right)}^{-1}\left(Y-X\beta -Z_{1}b_{1}-Z_{2}b_{2}\right)\right\}{\left(\sigma_{e}^{2}\right)}^{-\frac{\nu_{e}}{2}-1}{\left(\frac{\nu_{e}}{a_{e}}\right)}^{\frac{\nu_{e}}{2}}\text{exp}\left\{-\frac{\nu_{e}}{a_{e}}\frac{1}{\sigma_{e}^{2}}\right\}\propto {\left(\sigma_{e}^{2}\right)}^{-\frac{\nu_{e}+n}{2}-1}\text{exp}\left\{-\left(\frac{{\left(Y-X\beta -Z_{1}b_{1}-Z_{2}b_{2}\right)}^{T}\left(Y-X\beta -Z_{1}b_{1}-Z_{2}b_{2}\right)}{2}+\frac{\nu_{e}}{a_{e}}\right)\left(\frac{1}{\sigma_{e}^{2}}\right)\right\}\propto IG\left(\frac{\nu_{\epsilon }+n}{2},\frac{{\left(Y-X\beta -Z_{1}b_{1}-Z_{2}b_{2}\right)}^{T}\left(Y-X\beta -Z_{1}b_{1}-Z_{2}b_{2}\right)}{2}+\frac{\nu_{e}}{a_{e}}\right)$$

#### Variational posterior for $\sigma_{e}^{2}$

$$q\left(\sigma_{e}^{2}|ELSE\right)\propto \text{exp}\left\{E_{-\sigma_{\epsilon }^{2}}\left[\text{log}P\left(\sigma_{e}^{2}|ELSE\right)\right]\right\}\propto \frac{1}{{\left(\sigma_{e}^{2}\right)}^{\frac{\nu_{e}+1+n+1}{2}}}\text{exp}\left(-\frac{1}{\sigma_{e}^{2}}\left\{E_{-\sigma_{e}^{2}}\left(\frac{{\left(Y-X\beta -Z_{1}b_{1}-Z_{2}b_{2}\right)}^{T}\left(Y-X\beta -Z_{1}b_{1}-Z_{2}b_{2}\right)}{2}+\frac{\nu_{e}}{a_{e}}\right)\right\}\right)\propto \frac{1}{{\left(\sigma_{e}^{2}\right)}^{\frac{\nu_{e}+1+n+1}{2}}}\text{exp}\left(-\frac{1}{\sigma_{e}^{2}}\left\{\frac{e^{*T}e^{*}+tr\left(X^{t}X\Sigma_{q\left(\beta \right)}\right)+tr\left(Z_{1}^{T}Z_{1}\Sigma_{q\left(b_{1}\right)}\right)+tr\left(Z_{2}^{T}Z_{2}\Sigma_{q\left(b_{1}\right)}\right)}{2}+\nu_{e}\mu_{q\left(1/a_{e}\right)}\right\}\right)\propto IG\left(k_{q\left(\sigma_{e}^{2}\right)}=\frac{\nu_{e}+n}{2},B_{q\left(\sigma_{e}^{2}\right)}=\frac{e^{*T}e^{*}+tr\left(X^{t}X\Sigma_{q\left(\beta \right)}\right)+tr\left(Z_{1}^{T}Z_{1}\Sigma_{q\left(b_{1}\right)}\right)+tr\left(Z_{2}^{T}Z_{2}\Sigma_{q\left(b_{1}\right)}\right)}{2}+\nu_{e}\mu_{q\left(1/a_{e}\right)}\right)$$

with $e^{*}=Y-X\mu_{q\left(\beta \right)}-Z_{1}\mu_{q\left(b_{1}\right)}-Z_{2}\mu_{q\left(b_{2}\right)}$

#### Full conditional for $a_{e}$

$$P\left(a_{e}|ELSE\right)\propto P\left(\sigma_{e}^{2}|a_{e}\right)P\left(a_{e}\right)\propto {\left(\sigma_{e}^{2}\right)}^{-\frac{\nu_{e}}{2}-1}{\left(\frac{\nu_{e}}{a_{e}}\right)}^{\frac{\nu_{e}}{2}}\text{exp}\left\{-\frac{\nu_{e}}{a_{e}}\frac{1}{\sigma_{e}^{2}}\right\}{\left(a_{e}\right)}^{-\frac{1}{2}-1}{\left(\frac{1}{A_{e}^{2}}\right)}^{\frac{1}{2}}\text{exp}\left\{-\frac{1}{A_{e}^{2}}\frac{1}{a_{e}}\right\}\propto {\left(a_{e}\right)}^{-\frac{\nu_{e}+1}{2}-1}\text{exp}\left\{-\left(\frac{\nu_{e}}{\sigma_{e}^{2}}+\frac{1}{A_{e}^{2}}\right)\left(\frac{1}{a_{e}}\right)\right\}\propto IG\left(\frac{\nu_{e}+1}{2},\frac{\nu_{e}}{\sigma_{e}^{2}}+\frac{1}{A_{e}^{2}}\right)$$

#### Variational posterior for $a_{e}$

$$P\left(a_{e}|ELSE\right)\propto \text{exp}\left\{E_{-a_{e}}\left[\text{logP}\left(a_{e}|ELSE\right)\right]\right\}\propto \frac{1}{{\left(a_{e}\right)}^{\frac{\nu_{e}+1}{2}+1}}\text{exp}\left(-\frac{1}{a_{e}}E_{-a_{e}}\left(\frac{1}{A_{e}^{2}}+\nu_{e}\sigma_{e}^{-2}\right)\right)\propto IG\left(\frac{\nu_{e}+1}{2},B_{q\left(a_{e}\right)}=\frac{1}{A_{e}^{2}}+\nu_{e}\mu_{q\left(\sigma_{e}^{-2}\right)}\right)$$

## Appendix B

### Expression of the lower bound of the log likelihood for the variational Bayes model

$$\log P\left(y;q^{*}\right)=\int q\left(\theta \right)\log\left(\frac{P\left(y,\theta \right)}{q^{*}\left(\theta \right)}\right)d\theta =E_{q^{*}}\left[\log P\left(Y,\theta \right)-\log q^{*}\left(\theta \right)\right]=E_{q^{*}}\left[\log P\left(Y|\beta ,b_{1},b_{2},\sigma_{e}^{2}\right)\right]+E_{q^{*}}\left[\log P\left(\beta |\sigma_{\beta }^{2}\right)-\log q^{*}\left(\beta \right)\right]+E_{q^{*}}\left[\text{log}P\left(\sigma_{\beta }^{2}|a_{\beta }\right)-\log q^{*}\left(\sigma_{\beta }^{2}\right)\right]+E_{q^{*}}\left[\text{log}P\left(a_{\beta }\right)-\log q^{*}\left(a_{\beta }\right)\right]+E_{q^{*}}\left[\log P\left(b_{1}|\sigma_{1}^{2}\right)-\log q^{*}\left(b_{1}\right)\right]+E_{q^{*}}\left[\log P\left(b_{2}|\sigma_{2}^{2}\right)-\log q^{*}\left(b_{2}\right)\right]+E_{q^{*}}\left[\log P\left(\sigma_{1}^{2}|a_{b1}\right)-\log q^{*}\left(\sigma_{1}^{2}\right)\right]+E_{q^{*}}\left[\log P\left(a_{b1}\right)-\log q^{*}\left(a_{b1}\right)\right]+E_{q^{*}}\left[\log P\left(\sigma_{2}^{2}|a_{b2}\right)-\log q^{*}\left(\sigma_{2}^{2}\right)\right]+E_{q^{*}}\left[\log P\left(a_{b2}\right)-\log q^{*}\left(a_{b2}\right)\right]+E_{q^{*}}\left[\log P\left(\sigma_{e}^{2}|a_{e}\right)-\log q^{*}\left(\sigma_{e}^{2}\right)\right]+E_{q^{*}}\left[\log P\left(a_{e}\right)-\log q^{*}\left(a_{e}\right)\right]$$ (B1)

The first term in (B1) is

$$E_{q^{*}}\left[\log P\left(Y|\beta ,b_{1},b_{2},\sigma_{e}^{2}\right)\right]=-\frac{n}{2}\text{log}\left(2\pi \right)-\frac{n}{2}E_{q^{*}}\text{log}\left(\sigma_{e}^{2}\right)-\frac{1}{2}\left[{\left(Y-X\mu_{q\left(\beta \right)}-\Sigma_{h=1}^{2}Z_{h}\mu_{q\left(b_{h}\right)}\right)}^{T}\left(I_{n}\mu_{q\left(\sigma_{e}^{-2}\right)}\right)\left(Y-X\mu_{q\left(\beta \right)}-\Sigma_{h=1}^{2}Z_{h}\mu_{q\left(b_{h}\right)}\right)\right]-\frac{1}{2}tr\left(\left[I_{n}\mu_{q\left(\sigma_{e}^{-2}\right)}\right]X\Sigma_{q\left(\beta \right)}X^{T}\right)-\frac{1}{2}\Sigma_{h=1}^{2}tr\left(\left[I_{n}\mu_{q\left(\sigma_{e}^{-2}\right)}\right]Z_{h}\Sigma_{q\left(b_{h}\right)}Z_{h}^{T}\right)$$

The second term in B1 is

Eq∗[logP(β|σβ2)−logq∗(β)]=12Eq∗log(|Σq(β)|)−12Eq∗log(|σβ2Σβ|)−12μq(1σβ2){μq(β)Σβ−1Tμq(β)+tr(Σβ−1Σq(β))}+I2

The third term in B1 is

Eq∗[logP(σβ2|aβ)−logq∗(σβ2)]=νβ2log(2νβ)−νβ2Eq∗log(aβ)+I2log(σβ2)+Bq(σβ2)∗∗μq(1σβ2)−νβ+I2Eq∗log(|Bq(σβ2)|),with Bq(σβ2)∗∗=μq(β)Σβ−1Tμq(β)+12tr(Σβ−1Σq(β))

The fourth term in B1 is

$$E_{q^{*}}\left[\text{logP}\left(a_{\beta }\right)-logq^{*}\left(a_{\beta }\right)\right]=\;\log\Gamma \left(\frac{\nu_{\beta }+1}{2}\right)-\log\Gamma \left(\frac{1}{2}\right)-\frac{1}{2}\log\left(A_{\beta }^{2}\right)+\left(\frac{\nu_{\beta }}{2}\right)E_{q^{*}}log\left(a_{\beta }\right)-\left(\frac{\nu_{\beta }+1}{2}\right)E_{q^{*}}log\left(\frac{1}{A_{\beta }^{2}}+\nu_{\beta }\mu_{q\left(\frac{1}{\sigma_{\beta }^{2}}\right)}\right)+\mu_{q\left(1/a_{\beta }\right)}\nu_{\beta }\mu_{q\left(\frac{1}{\sigma_{\beta }^{2}}\right)}$$

The fifth term in B1 is

$$E_{q^{*}}\left[logP\left(b_{1}|\sigma_{b1}^{2}\right)-logq^{*}\left(b_{1}\right)\right]=\frac{1}{2}\text{log}\left({\left|\Sigma \right|}_{q\left(b_{1}\right)}\right)-\frac{1}{2}E_{q^{*}}\text{log}\left(\left|G\sigma_{1}^{2}\right|\right)-\frac{1}{2}\mu_{q\left(b_{1}\right)}^{T}\left[G^{-1}\mu_{q\left(\sigma_{1}^{-2}\right)}\right]\mu_{q\left(b_{1}\right)}-\frac{1}{2}tr\left(G^{-1}\mu_{q\left(\sigma_{1}^{-2}\right)}]\Sigma_{q\left(b_{1}\right)}\right)+\frac{J}{2}$$

The sixth term in B1 is

$$E_{q^{*}}\left[logP\left(b_{2}|\sigma_{b2}^{2}\right)-logq^{*}\left(b_{2}\right)\right]=\frac{1}{2}\text{log}\left(\left|\Sigma_{q\left(b_{2}\right)}\right|\right)-\frac{1}{2}E_{q^{*}}\text{log}\left(\left|\left(I_{I}⊗G\right)\sigma_{2}^{2}\right|\right)-\frac{1}{2}\mu_{q\left(b_{2}\right)}^{T}\left[\left(I_{I}⊗G^{-1}\right)\sigma_{2}^{-2}\right]\mu_{q\left(b_{2}\right)}-\frac{1}{2}tr\left(\left(I_{I}⊗G^{-1}\right)\sigma_{2}^{-2}]\Sigma_{q\left(b_{2}\right)}\right)+\frac{IJ}{2}$$

The seventh term in B1 is

Eq∗[logP(σ12|ab1)−logq∗(σ12)]=νb12log(2νb1)−νb12Eq∗log(ab1)+J2log(σ12)+Bq(σ12)∗∗μq(1σ12)−νb1+J2Eq∗log(|Bq(σ12)|),with Bq(σ12)∗∗=μq(b1)G2−1Tμq(b1)+tr(G−1Σq(b1))

The eighth term in B1 is

$$E_{q^{*}}\left[\log P\left(a_{b1}\right)-logq^{*}\left(a_{b1}\right)\right]=\text{log}\Gamma \left(\frac{\nu_{b1}+1}{2}\right)-\text{log}\Gamma \left(\frac{1}{2}\right)-\frac{1}{2}\text{log}\left(A_{b1}^{2}\right)+\left(\frac{\nu_{b1}}{2}\right)E_{q^{*}}log\left(a_{b1}\right)-\left(\frac{\nu_{l}+1}{2}\right)\times E_{q^{*}}log\left(\frac{1}{A_{b1}^{2}}+\nu_{b1}\mu_{q\left(\sigma_{1}^{-2}\right)}\right)+\mu_{q\left(1/a_{b1}\right)}\nu_{b1}\mu_{q\left(\sigma_{1}^{-2}\right)}$$

The ninth term in B1 is

$$E_{q^{*}}\left[\text{logP}\left(\sigma_{2}^{2}|a_{b2}\right)-logq^{*}\left(\sigma_{2}^{2}\right)\right]=\frac{\nu_{b2}}{2}\text{log}\left(2\nu_{b2}\right)-\frac{\nu_{b2}}{2}E_{q^{*}}\text{log}\left(a_{b2}\right)+\frac{IJ}{2}\text{log}\left(\sigma_{2}^{2}\right)+B_{q\left(\sigma_{2}^{2}\right)}^{**}\mu_{q\left(\frac{1}{\sigma_{2}^{2}}\right)}-\frac{\nu_{b2}+IJ}{2}E_{q^{*}}\text{log}\left(\left|B_{q\left(\sigma_{2}^{2}\right)}\right|\right),\text{with}\;B_{q\left(\sigma_{2}^{2}\right)}^{**}=\mu_{q\left(b_{2}\right)}^{T}G_{2}^{-1}\mu_{q\left(b_{2}\right)}+tr\left(G_{2}^{-1}\Sigma_{q\left(b_{2}\right)}\right)$$

The tenth term in B1 is

$$E_{q^{*}}\left[\text{logP}\left(a_{b2}\right)-logq^{*}\left(a_{b2}\right)\right]=\text{log}\Gamma \left(\frac{\nu_{b2}+1}{2}\right)-\text{log}\Gamma \left(\frac{1}{2}\right)-\frac{1}{2}\text{log}\left(A_{b2}^{2}\right)+\left(\frac{\nu_{b2}}{2}\right)E_{q^{*}}log\left(a_{b2}\right)-\left(\frac{\nu_{b2}+1}{2}\right)E_{q^{*}}log\left(\frac{1}{A_{b2}^{2}}+\nu_{b2}\mu_{q\left(\sigma_{2}^{-2}\right)}\right)+\mu_{q\left(1/a_{b2}\right)}\nu_{b2}\mu_{q\left(\sigma_{2}^{-2}\right)}$$

The eleventh term in B1 is

$$E_{q^{*}}\left[logP\left(\sigma_{e}^{2}|a_{e}\right)-logq^{*}\left(\sigma_{e}^{2}\right)\right]=\frac{\nu_{e}}{2}\text{log}\left(2\nu_{e}\right)-\frac{\nu_{e}}{2}E_{q^{*}}\text{log}\left(a_{e}\right)+\frac{n}{2}\text{log}\left(\sigma_{e}^{2}\right)+B_{q\left(\sigma_{e}^{2}\right)}^{**}\mu_{q\left(\frac{1}{\sigma_{e}^{2}}\right)}-\frac{\nu_{\epsilon }+n}{2}E_{q^{*}}\text{log}\left(\left|B_{q\left(\sigma_{e}^{2}\right)}\right|\right)\;,\text{with}\;B_{q\left(\sigma_{e}^{2}\right)}^{**}=e^{*T}e^{*}+tr\left(X^{t}X\Sigma_{q\left(\beta \right)}\right)+tr\left(Z_{1}^{T}Z_{1}\Sigma_{q\left(b_{1}\right)}\right)+tr\left(Z_{2}^{T}Z_{2}\Sigma_{q\left(b_{1}\right)}\right)$$

The twelfth term in B1 is

$$E_{q^{*}}\left[log\text{P}\left(a_{e}\right)-logq^{*}\left(a_{e}\right)\right]=\text{log}\Gamma \left(\frac{\nu_{e}+1}{2}\right)-\text{log}\Gamma \left(\frac{1}{2}\right)-\frac{1}{2}\text{log}\left(A_{e}^{2}\right)+\left(\frac{\nu_{e}}{2}\right)E_{q^{*}}log\left(a_{e}\right)-\left(\frac{\nu_{e}+1}{2}\right)\times E_{q^{*}}log\left(\frac{1}{A_{e}^{2}}+\nu_{e}\mu_{q\left(\sigma_{e}^{-2}\right)}\right)+\mu_{q\left(1/a_{e}\right)}\nu_{e}\mu_{q\left(\sigma_{e}^{-2}\right)}$$

## Appendix C

### Alternative version of the algorithm for implementing the VBME

For the VBME algorithm given here is an alternative version that ignores the priors of the components $B_{q\left(\sigma_{\beta }^{2}\right)},B_{q\left(a_{\beta }\right)}$ since the variance component $\sigma_{\beta }^{2}$ was fixed. Also, the components $B_{q\left(a_{b1}\right)}$, $B_{q\left(a_{b2}\right)},$ $B_{q\left(a_{e}\right)}$ were ignored since the components $\nu_{b_{1}}\mu_{q\left(\frac{1}{ab_{1}}\right)}$, $\nu_{b_{2}}\mu_{q\left(1/a_{b_{2}}\right)}$ and $\nu_{e}\mu_{q\left(\frac{1}{a_{e}}\right)}$ were replaced by the hyperparameters Sb1, Sb2 and Sbe. To provide informative priors for these parameters, you can use Sb1=0.25*var(y)*$\left(\nu_{b_{1}}+1\right)$, Sb2=0.25*var(y)*$\left(\nu_{b_{2}}+1\right)$ and Se= 0.5*var(y)*$\left(\nu_{e}+1\right)$.

Initialize: $\mu_{q\left(\beta \right)}=0$**,** $\mu_{q\left(b_{1}\right)}=0$**,** $\mu_{q\left(b_{2}\right)}=0$**,** $\Sigma_{q\left(b_{1}\right)}=I_{J}$, $\sigma_{\beta }^{2}=10,000$ $\Sigma_{q\left(b_{2}\right)}=I_{IJ}$, $B_{q\left(\sigma_{1}^{2}\right)},B_{q\left(\sigma_{2}^{2}\right)},B_{q\left(\sigma_{e}^{2}\right)}>0$.

Cycle:

$$\Sigma_{q\left(\beta \right)}\leftarrow {\left(\Sigma_{\beta }^{-1}\sigma_{\beta }^{-2}+X^{T}\left(I_{n}\mu_{q\left(\sigma_{e}^{-2}\right)}\right)X\right)}^{-1}$$

$$\mu_{q\left(\beta \right)}\leftarrow \Sigma_{q\left(\beta \right)}\left(\Sigma_{\beta }^{-1}\beta_{\beta }\sigma_{\beta }^{-2}+X^{T}\left[I_{n}\mu_{q\left(\sigma_{e}^{-2}\right)}\right]\left[Y-\sum_{h=1}^{2}Z_{h}\mu_{q\left(b_{h}\right)}\right]\right)$$

$$\Sigma_{q\left(b_{1}\right)}\leftarrow {\left(G^{-1}\mu_{q\left(\sigma_{1}^{-2}\right)}+Z_{1}^{T}\left[I_{n}\mu_{q\left(\sigma_{e}^{-2}\right)}\right]Z_{1}\right)}^{-1}$$

$$\mu_{q\left(b_{1}\right)}\leftarrow \Sigma_{q\left(b_{1}\right)}\left(Z_{1}^{T}\left[I_{n}\mu_{q\left(\sigma_{e}^{-2}\right)}\right]\left[Y-X\mu_{q\left(\beta \right)}-Z_{2}\mu_{q\left(b_{2}\right)}\right]\right)$$

$$\Sigma_{q\left(b_{2}\right)}\leftarrow {\left(G_{2}^{-1}\mu_{q\left(\sigma_{2}^{-2}\right)}+Z_{2}^{T}\left[I_{n}\mu_{q\left(\sigma_{e}^{-2}\right)}\right]Z_{2}\right)}^{-1}$$

$$\mu_{q\left(b_{2}\right)}\leftarrow \Sigma_{q\left(b_{2}\right)}\left(Z_{2}^{T}\left[I_{n}\mu_{q\left(\sigma_{e}^{-2}\right)}\right]\left[Y-X\mu_{q\left(\beta \right)}-Z_{1}\mu_{q\left(b_{1}\right)}\right]\right)$$

$$B_{q\left(\sigma_{1}^{2}\right)}\leftarrow \frac{\mu_{q\left(b_{1}\right)}^{T}G^{-1}\mu_{q\left(b_{1}\right)}+tr\left(G^{-1}\Sigma_{q\left(b_{1}\right)}\right)}{2}+Sb1$$

$$B_{q\left(\sigma_{2}^{2}\right)}\leftarrow \frac{\mu_{q\left(b_{2}\right)}^{T}G_{2}^{-1}\mu_{q\left(b_{2}\right)}+tr\left(G_{2}^{-1}\Sigma_{q\left(b_{2}\right)}\right)}{2}+Sb2$$

$$B_{q\left(\sigma_{e}^{2}\right)}\leftarrow \frac{e^{*T}e^{*}+tr\left(X^{t}X\Sigma_{q\left(\beta \right)}\right)+tr\left(Z_{1}^{T}Z_{1}\Sigma_{q\left(b_{1}\right)}\right)+tr\left(Z_{2}^{T}Z_{2}\Sigma_{q\left(b_{1}\right)}\right)}{2}+Sbe$$

until the change in the lower bound, $log\underline{p}\left(y;q\right),$ between iterations is less than a tolerance value specified a priori. In this case, the corresponding $\log\left(\underline{p}\left(y;q\right)\right)$ has the following analytical expression:

$$\text{log}\underline{p}\left(y;q\right)=0.5\log\left(\left|\Sigma_{q\left(\beta \right)}\right|\right)+0.5\log\left(\left|\Sigma_{q\left(b_{1}\right)}\right|\right)+0.5\log\left(\left|\Sigma_{q\left(b_{2}\right)}\right|\right)-0.5\left(\nu_{b1}+J\right)\log\left(\left|B_{q\left(\sigma_{1}^{2}\right)}\right|\right)-0.5\left(\nu_{b2}+IJ\right)\log\left(\left|B_{q\left(\sigma_{2}^{2}\right)}\right|\right)-0.5\left(\nu_{e}+n\right)\log\left(\left|B_{q\left(\sigma_{e}^{2}\right)}\right|\right)$$

## Appendix D

### R Code for Implementing the VBME Model

#####Set the working directory##################################################

rm(list=ls())

setwd(“C:\\Variationa Bayes Version\\VarBayes.Univariate”)

####Data###########################

load(“DATASET1.Wheat_GY.RData”)

Env1=rep(1,599)

Env2=rep(2,599)

Env3=rep(3,599)

Env4=rep(4,599)

Entry=c(1:599)

Env.All=c(Env1,Env2,Env3,Env4)

Entry.All=c(Entry,Entry,Entry,Entry)

YY.N=matrix(Y,ncol=1,byrow=F)

Hybrids=cbind(Env.All,Entry.All,c(YY.N+0))

colnames(Hybrids)=c(“Env”,”Entry”,”y”)

Hyb=as.data.frame(Hybrids)

##########Matrix of incidences#####################

X=model.matrix(∼0+as.factor(Hyb$Env))

Z1=model.matrix(∼0+as.factor(Hyb$Entry))

svda<-svd(G)

D<-diag(svda$d)

d_Inv=(1/svda$d)

D_Inv<-diag(d_Inv)

U<-svda$u

tV<-t(svda$v)

LL=U

Z1=Z1%*%LL

Gg=D

Z2=model.matrix(∼0+Z1:as.factor(Hyb$Env))

y=Hyb$y

betav=c(colMeans(Y))

tX=t(X)

tZ1=t(Z1)

tZ2=t(Z2)

I=ncol(X)

n=nrow(X)

J=ncol(Z1)

S_invB=diag(I)

d2_Inv=rep(d_Inv,I)

SigmaB=Sigmab1=Sigmab2=0.2

Sig.e=0.1

vb0=vb1=vb2=ve=2

data.set<-data.frame(Hyb)

K=1

####Lower bound of the log of the marginal likelihood (logp(y;q*)################

logpyq<- function(y,Mu.q.b0,Mu.q.b1,Mu.q.b2,Sig.q.b0,Sig.q.b1,Sig.q.b2,Bq.Sig.b0,Bq.Sig.b1,Bq.Sig.b2,Bq.Sig.be,X,Z1,Z2,Gg,vb0,vb1,vb2,ve){

n.T=length(y)

J=dim(Gg)[1] #############Number of lines#######################################

I=dim(X)[2] ############Number of environments######################################

K=1 ######Number of replications######################################

Inv.Gg=Gg

###############Expected values##################################################

Mu.q.inv.sig.be=(0.5*(ve+n.T))/Bq.Sig.be

Mu.q.inv.sig.b0=(0.5*(vb0+I))/Bq.Sig.b0

Mu.q.inv.sig.b1=(0.5*(vb1+J))/Bq.Sig.b1

Mu.q.inv.sig.b2=(0.5*(vb2+I*J))/Bq.Sig.b2

Inv.IG.D2=diag(d2_Inv)

tX=t(X)

####Lower bound of the log of the marginal likelihood (logp(y;q*)################

log.lik=0.5*log(det(Sig.q.b0))+0.5*log(det(Sig.q.b1))+0.5*sum(log(diag(Sig.q.b2)))-0.5*(vb1+J)*log(Bq.Sig.b1)-0.5*(vb2+I*J)*log(Bq.Sig.b2)-0.5*(ve+n.T)*log(Bq.Sig.be)}

YMatrix=matrix(y,ncol=4,byrow=F)

beta_zero=c(apply(YMatrix,2,mean,na.rm=TRUE))

Mu.q.b0.sv=rep(1,I)

Mu.q.b1.sv=rep(0.1,J)

Mu.q.b2.sv=rep(0.1,I*J)

Sig.q.b0.sv=diag(I)

Sig.q.b1.sv=diag(J)

Sig.q.b2.sv=diag(I*J)

Bq.Sig.b0.sv=Bq.Sig.b1.sv=Bq.Sig.b2.sv=Bq.Sig.be.sv=1

vb1=vb2=ve=5

Sb1=0.25*var(y)*(vb1+1)

Sb2=0.25*var(y)*(vb2+1)

Se=var(y-X%*%beta_zero)*(ve+1)

Inv.Gg=D_Inv

n.T=length(y)

tX=t(X)

tZ1=t(Z1)

tZ2=t(Z2)

LL0=logpyq(y=y,Mu.q.b0=Mu.q.b0.sv,Mu.q.b1=Mu.q.b1.sv,Mu.q.b2=Mu.q.b2.sv,Sig.q.b0=Sig.q.b0.sv,Sig.q.b1=Sig.q.b1.sv,Sig.q.b2=Sig.q.b2.sv,Bq.Sig.b0=Bq.Sig.b0.sv,Bq.Sig.b1=Bq.Sig.b1.sv,Bq.Sig.b2=Bq.Sig.b2.sv,Bq.Sig.be=Bq.Sig.be.sv,X=X,Z1=Z1,Z2=Z2,Gg=D,vb0=vb0,vb1=vb1,vb2=vb2,ve=ve)

LL0

tolerance<-c(0.00001) # Tolerance to declare convergence

converged=FALSE

maxIter=1000

itnum=0

while(!converged){

itnum=itnum+1;

######Updating the parameters of interest########################################

if (itnum==1) previous.LL0<-LL0

if (itnum>1) previous.LL0<-current.LL0

Mu.q.inv.sig.be=(0.5*(ve+n.T))/(Bq.Sig.be.sv)

Mu.q.inv.sig.b0=1/10000

Sig.q.b0=solve((diag(I))*Mu.q.inv.sig.b0+Mu.q.inv.sig.be*(tX%*%X))

Mu.q.b0=Sig.q.b0%*%(Mu.q.inv.sig.be*(tX%*%(y-Z1%*%Mu.q.b1.sv-Z2%*%Mu.q.b2.sv))+((diag(I))*Mu.q.inv.sig.b0)%*%beta_zero)

Mu.q.inv.sig.b1=(0.5*(vb1+J))/Bq.Sig.b1.sv

DDD1=1/(d_Inv*Mu.q.inv.sig.b1+Mu.q.inv.sig.be*I)

Sig.q.b1=diag(DDD1)

Mu.q.b1=Sig.q.b1%*%(Mu.q.inv.sig.be*tZ1%*%(y-X%*%Mu.q.b0-Z2%*%Mu.q.b2.sv))

Mu.q.inv.sig.b2=(0.5*(vb2+I*J))/Bq.Sig.b2.sv

d22_Inv=c(d2_Inv)

DDD2=1/(d22_Inv*Mu.q.inv.sig.b2+Mu.q.inv.sig.be)

Sig.q.b2=diag(DDD2)

Mu.q.b2=Sig.q.b2%*%(Mu.q.inv.sig.be*tZ2%*%(y-X%*%Mu.q.b0-Z1%*%Mu.q.b1))

Bq.Sig.b1=0.5*(t(Mu.q.b1)%*%(Inv.Gg)%*%Mu.q.b1+sum(diag(Inv.Gg%*%Sig.q.b1)))+Sb1

Inv.IG.D2=d22_Inv

Bq.Sig.b2=0.5*(t(Mu.q.b2)%*%(diag(Inv.IG.D2))%*%Mu.q.b2 +sum(diag(diag(Inv.IG.D2)%*%Sig.q.b2)))+Sb2

ym0=(y-X%*%Mu.q.b0-Z1%*%Mu.q.b1-Z2%*%Mu.q.b2)

Bq.Sig.be=0.5*(t(ym0)%*%(ym0)+sum(diag((tX%*%X)%*%Sig.q.b0))+sum(diag(I*Sig.q.b1))+sum(diag(Sig.q.b2))+Se)

####Lower bound of the log of the marginal likelihood (logp(y;q*)################

log.lik=0.5*log(det(Sig.q.b0))+0.5*(sum(log(DDD1)))+0.5*(sum(log(DDD2)))

-0.5*(vb1+J)*log(Bq.Sig.b1)-0.5*(vb2+I*J)*log(Bq.Sig.b2)-0.5*(ve+n.T)*log(Bq.Sig.be)

current.LL0<-log.lik

relErr<-abs((current.LL0/previous.LL0)-1)

if (itnum>=maxIter) {

converged=TRUE

print(“Warning:maximun number of iterations exceeded.”)

}

if(relErr<tolerance) converged=TRUE

Bq.Sig.be.sv=c(Bq.Sig.be)

Mu.q.b1.sv=Mu.q.b1

Mu.q.b2.sv=Mu.q.b2

Bq.Sig.b1.sv=c(Bq.Sig.b1)

Bq.Sig.b2.sv=c(Bq.Sig.b2)

##############Estimates of variance components##################################

Sig.b1=(Bq.Sig.b1)/(0.5*(vb1+J-2))

Sig.b2=(Bq.Sig.b2)/(0.5*(vb2+I*J-2))

Sig.e=(Bq.Sig.be)/(0.5*(ve+n.T-2))

cat(’Iteration:’, itnum, ’\n\n’)

cat(’Current LL0: ’,round(current.LL0,3), ’\n’, ’Previous LL0: ’,

round(previous.LL0,3), ’\n’, ’Tolerance: ’, round(relErr,3), ’\n’,

’Converged: ’, converged, ’\n\n’)

}

###########Printing parameter estimates#########################################

Par.estimates=rbind(Mu.q.b0,Sig.b1,Sig.b2,Sig.e)

Par.estimates
